# Randomised-controlled feasibility study evaluating the REgulate your SItting Time (RESIT) intervention for reducing sitting in individuals with type 2 diabetes: a process evaluation

**DOI:** 10.1136/bmjopen-2025-101309

**Published:** 2026-02-16

**Authors:** Stuart J H Biddle, Marsha L Brierley, Ellen Castle, Emily R Hunt, Angel Chater, Charlotte Edwardson, Daniel Bailey

**Affiliations:** 1 Centre for Health Research & Manna Institute, University of Southern Queensland, Springfield Central, UK; 2 Faculty of Sport & Health Sciences, University of Jyväskylä, Jyväskylä, Finland; 3 Department of Sport, Health and Exercise Sciences, Brunel University of London, London, UK; 4 Centre for Physical Activity in Health and Disease, College of Health, Medicine and Life Sciences, Brunel University of London, London, UK; 5 Department of Health Sciences, Brunel University of London, London, UK; 6 Institute for Sport and Physical Activity Research, University of Bedfordshire, Bedford, UK; 7 Centre for Behaviour Change, University College London, London, England, UK; 8 Leicester Lifestyle and Health Research Group, Diabetes Research Centre, University of Leicester, Leicester, UK; 9 NIHR Leicester Biomedical Research Centre, Leicester General Hospital, Leicester, UK

**Keywords:** Exercise, Diabetes Mellitus, Type 2, SPORTS MEDICINE, Behavior, Digital Technology

## Abstract

**Objectives:**

The REgulate your SItting Time (RESIT) is a tailored intervention targeting reductions and breaks in sitting in adults with type 2 diabetes mellitus (T2DM). A feasibility trial of RESIT had been conducted and the purpose of this paper is to report findings from the process evaluation.

**Design:**

A mixed-methods process evaluation within a randomised controlled feasibility trial.

**Setting:**

The study was conducted remotely in the community.

**Participants:**

Ambulatory individuals with T2DM aged 18–85 years.

**Intervention:**

A tailored intervention comprising an online education session, regular health coaching and technology for self-monitoring behaviour and prompting breaks in sitting.

**Primary and secondary outcome measures:**

Questionnaires (intervention participants n=22 at both 3 and 6 months; control participants n=21 at 3 months, n=29 at 6 months) and interviews (n=30, with n=13 intervention participants, n=12 control participants, n=5 health coaches) to assess perceptions of the intervention components, strategies and barriers for sitting less, the role of the study evaluation measures, and reasons for taking part.

**Results:**

The trial operated a largely successful online education element for those in the intervention group (82% completion; ≥76% engagement in individual educational elements). There was good use of self-monitoring and prompt technology (apps and wearables) with 73% of participants reporting using these at 6 months. Health coaching had high engagement and was perceived as enjoyable and useful. Data revealed strategies used for behaviour change (eg, active functional tasks) alongside barriers to change (eg, restrictions at work). There were also potential behavioural influences from the study evaluation measures (eg, activity measures increasing awareness and execution of behaviours) for both intervention and control participants.

**Conclusions:**

A comprehensive process evaluation identified successful intervention elements (ie, online education, health coaching, wearables and smartphone apps) alongside strategies and barriers to behaviour change. These findings can inform future sedentary behaviour interventions for adults with T2DM and a definitive randomised controlled trial evaluating RESIT.

**Trial registration number:**

ISRCTN14832389.

**STRENGTHS AND LIMITATIONS OF THIS STUDY**

- A comprehensive mixed-methods process evaluation was conducted that has strengthened the learnings from this feasibility trial.
- Qualitative and quantitative data were integrated to provide an in-depth understanding of factors affecting trial and intervention implementation.
- The quantitative data analysis was limited to the participants who responded to the process evaluation questionnaires, which could influence the findings.

## Introduction

 Higher levels of sedentary behaviour—sitting/lying with low energy expenditure during waking hours^1^—are adversely associated with cardiometabolic health, glycaemia, depression and quality of life in individuals with type two diabetes mellitus (T2DM).^2^,^6^ In contrast, regularly breaking up sitting with standing, walking and simple resistance exercises results in improved cardiometabolic biomarkers over a single day in individuals with T2DM and impaired glycaemia.^7^,^9^ Recommendations for management of T2DM, therefore, include a focus on reducing and breaking up sedentary behaviour.^10 11^

Interventions targeting reductions in sedentary behaviour have often focused on the workplace due to the usual necessity to work at a computer while sitting.^12^ Although these interventions have been successful and informative with regards to the active ingredients that bring about behaviour change,^13 14^ their generalisability to non-work contexts and individuals with long-term health conditions is unclear. The REgulate your SItting Time (RESIT) intervention was developed to address the lack of interventions supporting individuals with T2DM to reduce and break up sitting. A randomised controlled feasibility trial was undertaken to assess the feasibility and acceptability of delivering and evaluating the RESIT intervention in ambulatory individuals with T2DM. This is described fully in a published protocol by Bailey *et al*.^15^ Briefly, participants were individually randomised to an intervention or control group. The intervention group received the multicomponent RESIT intervention, which was designed to reduce sitting throughout the entire waking day. The intervention was delivered remotely and included an interactive online education session, health coach support (sessions at intervention start and 2, 6 and 12 weeks) and a choice of wearables, smartphone apps and computer software that supported self-monitoring of sitting, feedback and prompts to break up periods of sedentary behaviour. The control group participants continued with their usual healthcare. Results indicated that the intervention and evaluation methods were feasible and acceptable to participants.^16^ There were also indications that the intervention had potential for reducing daily and prolonged sitting and improving health and well-being.^16^

An important element of any randomised controlled trial (RCT), especially those considered ‘complex interventions’,^17^ is a process evaluation. This provides information concerning implementation (eg, fidelity), possible reasons for outcomes (eg, mechanisms of impact) and contextual factors (eg, world events) shaping intervention outcomes.^18^ To better inform implementation and future intervention research, it is important to understand the experience and engagement of participants with different intervention components, as well as the intervention overall. Understanding what works, why, for whom and in what context is critical knowledge for interventions targeting T2DM. Therefore, to better understand how the RESIT intervention operated and was perceived by participants, in addition to reasons for participating and experiences of taking part, we undertook process evaluation assessments with intervention and control group participants at 3-month and 6-month follow-ups, and interviews with health coaches who were involved in delivering the intervention. Intervention acceptability, fidelity and engagement are reported elsewhere alongside outcomes related to feasibility and acceptability of the trial.^16^

The aim of this paper is to report the process evaluation findings to understand, in depth, how a tailored intervention (RESIT) to reduce and break up sitting in individuals with T2DM operated and was experienced during a randomised controlled feasibility trial.

## Methods

### Study design

The methods of the randomised controlled feasibility trial are described fully in a previous publication by Bailey *et al*.^15^ The trial protocol is available in online supplemental file 1. In brief, participants were ambulatory individuals with T2DM who were individually randomised to receive either the intervention (RESIT) or control (usual care) for 6 months. The process evaluation employed a mixed-methods approach with data collected via questionnaires and semi-structured individual interviews with both intervention and control participants. The health coaches involved with the delivery of RESIT were also interviewed. The data collected from the study participants and methods used are shown in table 1. The study took place from October 2020 to November 2021 in England; hence, it was conducted online during the COVID-19 pandemic with lockdown restrictions often in place.

**Table 1:** Process evaluation methods and data collected for intervention and control participants

	Intervention participants			Control participants		
	3-month questionnaire	6-month questionnaire	Interview	3-month questionnaire	6-month questionnaire	Interview
Online education session						
Perceptions of content	√	√	√			
Increased awareness and motivation	√	√	√			
Key messages	√	√	√			
Impact on behaviour	√	√	√			
Wider dissemination	√	√	√			
Wearables (for goal setting/self-monitoring behaviour)						
Use	√	√	√			
Usefulness	√	√	√			
Improvements	√	√	√			
Strategies for use	√	√	√			
Usability of device	√	√	√			
Ease of use	√	√	√			
Smartphone apps						
Use	√	√	√			
Usefulness	√	√	√			
Improvements	√	√	√			
Strategies for use	√	√	√			
Usability of device	√	√	√			
Ease of use	√	√	√			
Computer software						
Use	√	√	√			
Usefulness	√	√	√			
Usability of device	√	√	√			
Ease of use	√	√	√			
Facilitated behaviour change	√	√	√			
Alternative support for self-monitoring and/or prompt						
Use of other self-monitoring or prompt tools	√	√	√			
Motivations for taking part and remaining in the study			√			√
Family, friends, colleagues support	√	√	√			
Strategies used to change sitting behaviour (wearable, smartphone app, computer software, goal setting, prompting, education)	√	√	√			
Behaviour changes resulting from participating in the study	√	√	√	√	√	√
Motivators for behaviour change	√	√	√			
Facilitators to behaviour change	√	√	√			
Potential for cross-contamination				√	√	√
Benefits of reducing sitting and negative/adverse events	√	√	√	√	√	√
Behaviour change maintenance	√	√	√			
Major life events	√	√	√	√	√	√
Other lifestyle changes (eg, nutrition, exercise)	√	√	√	√	√	√
Impact of study assessments on behaviour	√	√	√	√	√	√

The Consolidated criteria for Reporting Qualitative research (COREQ) checklist was used for appropriate parts of the report (see COREQ checklist and online supplemental table 1).

### Data collection

#### Questionnaires

For the process evaluation, questionnaires were provided to all intervention and control participants at 3-month and 6-month follow-up (see online supplemental table 2 for completion rates). The questionnaires were developed following Medical Research Council guidelines^18^ and informed by previous research. Forced choice, Likert scaled items and open-ended questions were included.^19^ Intervention group participants were asked questions concerning their engagement with, and experiences of, the online education and the use of wearables and apps. In addition, they answered questions about alternative support they may have used, strategies for sitting less and barriers to behaviour change.

Both intervention and control group participants were asked questions about any life-changing events that may have impacted on health-related behaviours, and any impact of the study measurements on their lifestyle or sitting time. Table 1 shows the focus of all questionnaire and interview items for the intervention and control participants.

#### Interviews

Semi-structured interviews were conducted by MLB online or by telephone. MLB was involved in the delivery of the project (participant recruitment, intervention facilitation and outcome measure evaluation). Participants (n=65) were invited at random to take part in the interviews following completion of their 6-month measurements. Of those, 45 agreed to interview (20 Control and 25 Intervention) with 31 booking an interview (15 Control, 16 Intervention) and 25 attending an interview (12 Control, 13 Intervention). We spoke with 37% of intervention participants (n=13 of 35 starting at baseline; 54% female; age 63±11 years) and 34% of controls (n=12 of 35; 50% female; age 57±10 years). Raw demographic characteristics of all interviewees have been reported previously by Brierley *et al*.^16^ Demographic characteristics of the participants interviewed were similar to the sample of their corresponding study arm (see online supplemental table 3). Interviews with the study participants lasted between 14 and 72 min. The interviews explored motivations for taking part in the study, behavioural changes made during the study and responses to taking part in the study measurements. Interviews with intervention participants additionally explored experiences with each of the RESIT components, including behaviour change strategies used and barriers encountered. All RESIT health coaches (n=5; 80% female) were interviewed. These interviews lasted between 22 and 43 min and explored overall perceptions of the RESIT intervention and their experiences with delivering the health coaching sessions.

### Data analysis

A convergent design of analysis and triangulation was used,^20^ in which the questionnaire and interview data was analysed separately, to capitalise on the expertise within the research team, and then integrated where appropriate.

For questionnaire items requiring responses on yes/no and Likert scaled items, descriptive statistics, including frequencies and percentages, were calculated using Microsoft Excel. Frequency and percentage data for each questionnaire item were calculated based on the number of participants who answered that item. Open-ended responses on the questionnaires were grouped into coherent themes using template analysis.^21^ All statements were entered in MindGenius (V.20) software and grouped by themes and subthemes.

The interviews were analysed as part of the wider randomised controlled feasibility trial.^16^ Interviews were recorded and transcribed verbatim using Otter AI (Otter.ai, Mountain View, California, USA). The transcripts were checked and corrected manually by the research team before being imported into NVivo V.12 (Lumivero, Denver, Colorado, USA) for support with data coding and organising. Key themes were identified using the Framework Method of Analysis.^22^ Stages 1–3 of the Framework Method were conducted independently by two researchers (not involved with any delivery aspects of the trial) to identify inductive codes (ERH) that were based on the data and deductive codes (EC) that were based on the trial protocol. The generated inductive and deductive data were combined, with equal weighting, to develop the agreed ‘working analytical framework’ (stage 4).^22^ Reflexivity and rigour were maintained throughout the analysis through reflexive journaling and discussions within the research team. The themes and subthemes from the interview analysis were then integrated with questionnaire data when interpreting and writing the findings.^23^ For qualitative data reported previously regarding intervention acceptability and fidelity^16^ that also relates to the aims of the process evaluation (eg, understanding participant perceptions of the intervention), that data has been interpreted where relevant but without accompanying quotations. Data refer to participants rather than the health coaches, unless indicated.

### Patient and public involvement

Individuals with T2DM helped develop the research question, emphasising the importance of targeting reductions in sedentary behaviour as a distinct behaviour from physical activity. The intervention design was informed by public members who supported an approach for individual tailoring through options for different digital tools that supported the target behaviour. Public members also advised on strategies for recruiting participants and helped design public-facing study information. There was a public advisory group during the research, which supported the development and direction of the research, development of information resources and dissemination of findings.

## Results

**Figure 1:**
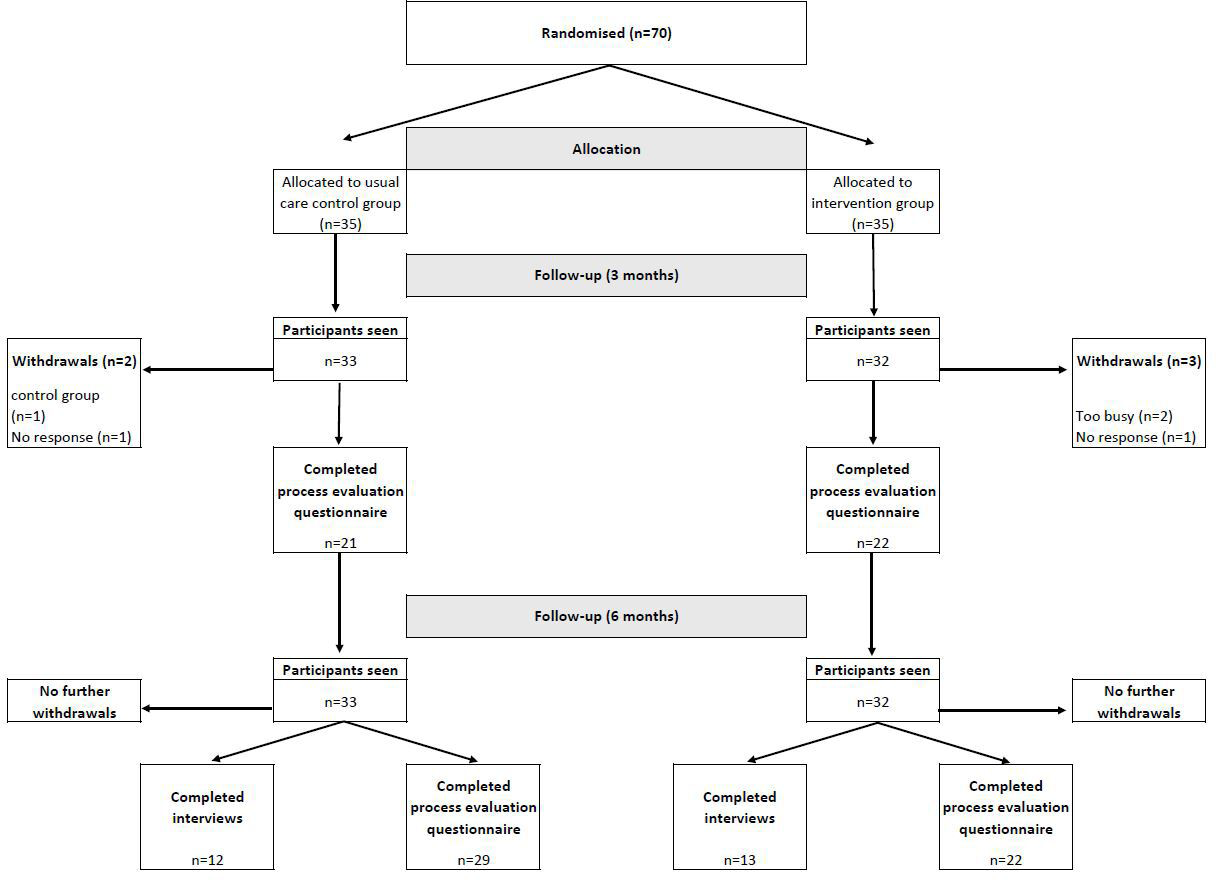
Flow of participants throughout the study.

A total of four themes were constructed from the interview data. The original thematic map alongside a subset of nested qualitative results is presented within the paper reporting on the feasibility and acceptability outcomes of the trial.^16^ The current paper focuses on the ‘reasons for participation’ and ‘outcomes of intervention’ themes. Results from the questionnaire data are reported for the intervention group in relation to (1) online education (assessed at 3 months only); (2) use of smartphone apps, computer software, wearable devices and alternative support; (3) strategies for sitting less; (4) barriers to sitting less; (5) other behavioural changes; and (6) the role of study assessments, with the latter two also reported by control participants. The flow of participants throughout the study is shown in figure 1. Results are for participants unless otherwise indicated.

### Reasons for participation

One key theme from the interview data concerned reasons participants expressed for participating in the study. Subthemes included health motivation, altruism and having the time.^16^ Health motivation reflected the desire to improve one’s health, such as diabetes and other long-term conditions. As one participant explained, “You’ve got to reach out for anything that is going to help you” (R07, intervention group (IG)). Another elaborated on how the study was an opportunity to further manage their diabetes:

Because I’ve had the diagnosis of type two diabetes … I’m just monitored, not on any medication. And I thought it would be a good idea to just have someone look at that and see if it can make changes - yes to help it. To help that condition. (RS02, control group (CG))

Other reasons for signing up to the study appeared to be altruism, to contribute to research and an opportunity to give back to the community: “I’ve had my life saved by health professionals, basically on two or maybe three occasions. So, I wanted to give something back” (RS04, IG). Research was valued by participants and seen to influence and facilitate improvements with care: “you know, this work might lead to something that gets implemented by the NHS, which could help millions of people” (RS60, CG).

Some participants reported that additional time, either related to retirement and/or working from home during the COVID-19 pandemic lockdown period, contributed to their availability for study participation. As voiced by one participant, “Part of the reason for doing this study if I was working, I wouldn’t have done it. It’s just having more time” (RS09, CG). Lockdown during the COVID-19 pandemic provided a unique opportunity for participants to take part in remote research like this study: “I think this started in the middle of lockdown or something like that. I thought it was a good moment to at least do something different from home” (RS25, CG).

### Online education

Of the intervention participants who responded to the questionnaire (63%; n=22), 82% (n=18) reported fully completing the online education session (online supplemental table 4). The level of the session was considered appropriate/easy to understand (86% agree/strongly agree and 90% agreed/strongly agreed that the session increased awareness of the health consequences of too much sitting). Nearly all participants (90%) agreed that the session motivated them to change their sitting time (table 2).

**Table 2:** Assessment of online education session components

	N	%*
Level was appropriate		
Strongly agree	4	19
Agree	14	67
Neither disagree nor agree	3	14
Disagree	0	0
Strongly disagree	0	0
Length was appropriate		
Strongly agree	4	19
Agree	14	67
Neither disagree nor agree	3	14
Disagree	0	0
Strongly disagree	0	0
Session increased awareness		
Strongly agree	12	57
Agree	7	33
Neither disagree nor agree	2	10
Disagree	0	0
Strongly disagree	0	0
Motivated me		
Strongly agree	7	33
Agree	12	57
Neither disagree nor agree	1	5
Disagree	1	5
Strongly disagree	0	0

* % is calculated as number of responses divided by number of participants that reported having completed (or partially completed) the online education session (n=21) multiplied by 100.

Most participants completed the worksheet to calculate their sitting time (86%), read the top tips for reducing sitting (95%), completed the goal-setting sheet (86%), with slightly fewer watching the animations (76%) (online supplemental table 5). The majority of participants considered these elements to be at least ‘moderately useful’ (85–100% across elements) (online supplemental table 6).

Concerning the online education, three main themes were identified from the process evaluation questionnaires: ‘moving more’, ‘regulation of sitting’ and ‘health messages’. For the ‘moving more’ theme, participants highlighted a number of beliefs and messages (eg, ‘to be more active’; ‘need to exercise’; ‘realisation of how inactive I am’) and strategies (eg, ‘move at regular intervals’; ‘more walking’; ‘exercise daily’). For the theme ‘regulation of sitting’, responses were grouped by subthemes of ‘sit less’ (eg, ‘reduce sitting time’), ‘stand more’ (eg, ‘it helps just to stand up for a few minutes’), ‘breaking up sitting’ (eg, ‘it’s important to get up and move around regularly’), ‘behavioural strategies’ (eg, ‘use my watch as a reminder’) and ‘sitting awareness and messages’ (eg, ‘how sitting affects my health’; ‘awareness of sitting time in my day’). Overall messages relating to moving more and reducing sitting were also identified from the interview data, despite participants having limited recall of the online education. Finally, five subthemes from the questionnaire data were identified within the theme of ‘health messages’. These included ‘diet’ (eg, ‘check what you eat’), ‘sleep’ (eg, ‘sleep health’), ‘psychology’ (eg, ‘not set too high a goal’; ‘be positive’), ‘general health messages’ (eg, ‘take care of yourself’) and ‘habit’ (eg, ‘small changes in habit will make a difference’).

### Wearable devices, smartphone apps and computer software

Three types of digital technology were available for use by the intervention group (consumer wearable activity trackers, smartphone apps and computer software; for selections, see online supplemental table 7). Most participants in the intervention group reported using at least one of these tools at 3 months (86%, declining to 73% at 6 months; online supplemental tables 8,9). Participants used one of the three consumer devices provided as part of the RESIT intervention (Xiaomi Mi Smart Band 4, Garmin Vivofit 4 or zTrack), or they used their own wearable. From the participants who used a wearable, 64% and 50% reported using it daily at 3 and 6 months, respectively. For smartphone apps, 45% and 36% reported daily use at 3 and 6 months, respectively. Computer software use was lower, with 18% and 0% reporting daily use at 3 and 6 months, respectively (online supplemental tables 10,11). The majority of participants found the wearables very or extremely useful for reminding them to break up sitting at 3 months (72%) and 6 months (76%) (table 3). The interview data supported this, with participants highlighting that the wearables and apps acted as a reminder if they had been sitting for too long, as reported previously by Brierley *et al*.^16^

**Table 3:** Usefulness of wearables, smartphone app(s) and computer software for reminding participants to break up sitting

	at 3 months		at 6 months	
	N	%*	N	%*
Wearables				
5-Extremely useful	8	44	7	41
4-Very useful	5	28	6	35
3-Moderately useful	3	17	0	0
2-Slightly useful	1	6	0	0
1-Not at all useful	1	6	4	24
Did not use	4	--	5	--
Smartphone apps				
5-Extremely useful	6	40	6	40
4-Very useful	5	33	4	27
3-Moderately useful	0	0	1	7
2-Slightly useful	1	7	2	13
1-Not at all useful	3	20	2	13
Did not use	7	--	7	--
Computer software				
5-Extremely useful	0	0	0	0
4-Very useful	2	29	0	0
3-Moderately useful	2	29	1	17
2-Slightly useful	0	0	0	0
1-Not at all useful	3	43	5	83
Did not use	15	--	16	--

* % is calculated as number of responses divided by number of participants providing a rating for that tool at each time point multiplied by 100.

While the majority of participants reported the usefulness of these tools for reducing and breaking up sitting, a minority reported not using a wearable (14% and 36% at 3 and 6 months, respectively) or smartphone app (32% and 55%, respectively) (online supplemental table 11). The wearable device and smartphone apps, more so than the computer software, were seen as encouraging in helping participants sit less (online supplemental table 12). Reasons given in the open-ended questionnaire responses for not using these tools included some of the app features not working, participants not using apps in general, participants managing without apps and wearables, and restricted access to a smartphone at work. It is possible that general disuse of wearables and apps may be related to fear of technology, as identified within the interview data relating to intervention fidelity.^16^

Participants in the intervention group were asked via questionnaire if they used any other devices, tools or methods not already in the RESIT intervention to support them in reducing their sitting. As online supplemental table 13 shows, most participants reported that they did not use alternative support.

### Health coaching

Participants engaged highly with the health coaching component of RESIT, with 113 of the 140 available sessions attended (81% attendance rate). In general, the health coaching was described by participants as a useful component of the intervention within the interviews. Usefulness typically referred to having social support from the health coach, with participants explaining that this was because they had someone to talk with, discuss options for behaviour change and receive encouragement. Participants often perceived the health coaches to be skilled in active listening, and this was considered to be important in helping them set goals. Similarly, several participants reported developing good rapport with the health coaches and felt listened to. One participant reported struggling to build rapport with the health coach and some participants highlighted that they wanted more time with the health coach to connect:

I didn’t particularly feel connected but I did connect. I did connect in those 15 minutes even. Okay, I was able to share what I wanted to. But if there was more time that would have been more appropriate”. (RS47, IG)

This suggestion was echoed by one health coach who suggested that the first session could be longer. Overall, participants spoke positively about the health coaching sessions, often describing them as enjoyable and interesting, which reflected good acceptability of the overall intervention.^16^ In relation to outcomes of the intervention, one participant explained how they perceived the health coaching to increase awareness of their sitting behaviour:

in my house actually, is a clock in every room (laughs). That actually, consciously, I think, consciously, was after a while, not immediately, but after a while, after your coaching, I started to look at the watch every now and then to see that half an hour has passed, so I should get up and do some exercises. (RS31, IG)

### Strategies and thoughts concerning sitting less

Intervention participants were asked in the questionnaire to list all of the strategies that they used to sit less at home and at work, with most using strategies in both environments. Strategies identified for the home environment, using template analysis, centred on ‘breaking up sitting’ (eg, creating standing tasks, standing for hobbies), ‘purposeful physical activity’ (eg, housework or walking in the garden) and ‘incidental activity’ (eg, different tasks, being reminded to move), with similar responses at 3 and 6 months. At work, similar themes were noted at 3 months, but at 6 months there were themes reflecting undertaking more work tasks that could be completed while standing, using prompts to help with reminders about breaking up sitting and identification of barriers to moving more at work. These findings from the open-ended responses were also confirmed from the interview data. Participants conveyed that an increased awareness of their sitting time contributed to reducing sitting throughout the day:

I definitely - I’m a lot more aware. I mean it’s not like the half an hour I get up, but I’m definitely up a lot more then I ever used to be. I could sit around for a whole day, and yeah, when I can’t, I don’t do that anymore. (RS14, IG)

For some, participants altered typical sedentary tasks to reduce sitting, such as when working on computers and speaking on the telephone:

When I’m working on computer, as I work for [charity name] as well. So, three days I usually sit down, but even then, having this study had an impact, because I try and make sitting time less while I was working on computer. (RS47, IG)

Several participants noted these types of modifications to sitting and were optimistic that these changes would lead to healthy habits:

‘It’s been very useful and I’m just hoping that having been on it for six months it will be a continuous thing rather than, you know? I’ve built a habit. (RS63, IG).

Making alterations to sedentary behaviour also extended to future plans beyond the study, as one health coach recalled:

…by the end of the programme he [IG participant] … was kind of thinking ahead, like, oh, you know, I’ve already booked because he had some flights coming up. I’ve made sure that I’ve booked an aisle seat so that we’ll be able to walk up and down like when the seatbelt sign is off. (health coach (HC)2)

Participants also described inventing reasons to sit less. For this, they would use active or functional tasks to break up or replace periods of sitting, such as cleaning, errands and changing routines to replace sitting with more active behaviours like standing and walking:

It then made me - my house was tidier (laughs). Because I would go ‘Right, Okay, I’m getting up and I’m going to have a cup of tea, but I’m standing in the kitchen while I’m in the kitchen’, you know? I would be conscious of what my body was actually doing. (RS07, IG)

So instead of straight to the post-box, which I’d have to have a reason I’d have to post something that I’ve walked round, wiggly route round and back home. To make it a bit more interesting. (RS11, IG).

These quotes portrayed the importance of task-oriented strategies to reduce sitting. Health coaches observed that participants often needed a purpose or reason to sit less. This was challenging for some participants to begin with, but became easier over time:

[a] lot of the time is taking a break from your desk at work and getting up to go to the loo or getting a coffee or chatting to a colleague, things like that. So those are all things to break up their sitting time. So, a lot of them found it kind of challenging at first too, because you’re so used to just sitting down and working away all day. But the more you kind of get these habits built into place, the more they become quite easy to follow up with after time. (HC1).

Intervention participants also described efforts to sit less by thinking about increasing engagement in structured physical activity. This included purchasing exercise equipment and signing up to exercise classes:

I loved the concept and I like the idea and you know, I’d get into it. And you know, I went out and bought a hula hoop, skipping rope, I was trying to do other activities. (RS07, IG)

I think I did mention I have started some Zoom classes for chair aerobics. (RS68, IG)

Occasionally, participants mentioned increasing incidental physical activity:

Sometimes I’ll just march on the spot. Sometimes I’ll perhaps go to the kitchen and do some work in the kitchen … I might walk up the stairs and walk down again. (RS63, IG).

The primary target behaviour for the RESIT intervention was reducing sitting, but participants sometimes had difficulty differentiating between sitting less and increasing planned physical activity. This may not be surprising given that physical activity can replace sitting. As one health coach remarked:

A lot of them [participants] did focus on trying to increase their activity levels. (HC1)

Participants’ thoughts, feelings and intentions (cognitions and affect) around sitting and physical activity were affected by the intervention. Often, participants expressed that sitting time was at the forefront of their mind and that this increased awareness led to behaviour change. One participant explained that their increased awareness of sitting time was linked to feelings of guilt, ultimately motivating them to sit less:

I think the primary influence was guilt. I think in a way that was the primary motivation, was seeing, oh god, I’ve been sitting for an hour and a half, particularly if you’re watching a film or something like that. Oh god, I’ve been sitting for, you know, I’ve been at the computer for half an hour, for an hour, so I had to, I had to make um, I had to make conscious ‘pit stops’, if you like. (RS49, IG).

Health coaches theorised that this increased awareness led to greater intentions to sit less:

it didn’t just change his actual habits to change his way of thinking as well. So, he was thinking ahead as to how he could incorporate his lifestyle needs I suppose into if he’s working. (HC2)

### Barriers to sitting less

Four main themes from the open-ended responses were identified at both 3 and 6 months relating to barriers to sitting less during the intervention: ‘work-related barriers’, ‘disability/health’, ‘fatigue/sleep’ and ‘context’. For work-related barriers, many participants had work to undertake on computers that were positioned without options to stand, with responses such as ‘meetings require me to be at a desk with a computer’, ‘the job is all computer work’ and ‘sometimes not possible to go away from screens’.

Some participants reported health and disability-related barriers, including hospital appointments, pain and illness. Sleepiness, particularly after lunch, and fatigue were also stated as barriers to sitting less. Finally, context was a theme that included social contextual barriers (eg, difficult to sit less with home visitors or when entertaining at home), and environmental contextual barriers (eg, weather/climate conditions).

### Other behavioural and context changes

Participants in the intervention group were asked via open-ended responses in the questionnaire whether anything had changed in their lives that might impact on their health-related behaviours (online supplemental table 14). At 3 months, 23% stated that this was the case, including changes in diet and spending extra time studying. At 6 months, these figures rose to 45%. Open-ended responses alluded to disruptions or illness due to the COVID-19 pandemic, family health issues and bereavements hindering healthy behaviour change, while some reported increases in exercise.

### Non-intervention influences on sitting and health behaviours

Relative to intervention participants, slightly fewer control participants reported changes in their health-related behaviours due to factors outside of the study (online supplemental table 15). At 3 months, questionnaire free-text responses from intervention participants mentioned the adoption of exercise, health issues, weight counselling and easing of COVID-19 lockdown restrictions. At 6 months, changes due to greater sport and exercise involvement, diet and moving house were mentioned by some.

Control group participants were also asked if they felt that participation in the study changed their sitting behaviours (online supplemental table 16). Just under one-third (29%) felt that they had made changes at 3 months, including greater awareness of sedentary behaviour and the need to move more. One participant mentioned that having a wearable device (not provided as part of the study) made a huge difference to their activity levels. At 6 months, 21% reported making changes, including using a sit-stand desk and Apple watch reminders.

The health assessments were perceived as positive for behaviour change by control and intervention participants (online supplemental table 17,18). Monitoring and outcome measures seemed to increase awareness of behaviour, as one participant expressed in the interviews: “Whenever I had the little monitor on [activPAL], you know, it always reminded me of the importance of standing up” (RS23, CG). One control participant reported the effect of having the measurements taken:

I think it makes you realise the size of your waist. You realise … the waist is not really that outlined. And then maybe made you think about movement and diet … I think it made me feel that my job and my lifestyle is very much quite sedentary. It made me move an awful lot more. (RS02, CG)

Although all participants did not receive their outcome results until the end of the study, having the outcome measured appeared to influence awareness and execution of behaviour change. This included awareness of the importance of healthy behaviours, such as reducing sedentary time, increasing physical activity and following a healthy diet. In contrast, others were seemingly not impacted by the measurements, as one participant outlined, “I don’t remember the measurements. I didn’t pay attention to it. I felt maybe it’s not as important” (RS25, CG).

## Discussion

We report on a process evaluation of a randomised controlled feasibility trial that delivered and evaluated a tailored intervention to reduce and break up sitting in adults with T2DM. Data included evaluation of the online education provision, digital technology support, health coaching, strategies and barriers concerning sitting behaviours, the role of study health assessments and reasons for participation. A range of important issues, in line with recommendations for process evaluations,^18 24^ has been identified that can be used to inform future definitive RCTs evaluating remotely delivered sedentary behaviour interventions for individuals with T2DM.

Intervention group participants showed good engagement with RESIT’s online education session. Completion of the session was high and engagement in the different sections was good. This is similar to prior studies, suggesting that both face-to-face^13^ and online education^25^ can be successfully adopted as a core component of behaviour change interventions. However, education alone may be insufficient. For example, education-based interventions typically have low-to-small effects,^26 27^ or no effect at all, including those targeting sedentary behaviour.^28^ Education may be important as an initial phase of behaviour change but will likely need additional behavioural strategies to sustain behaviour change. The additional behavioural strategies delivered within the RESIT multicomponent intervention may have been important in this respect.

All elements of the RESIT online education session had fairly high engagement, even for those that were more time-consuming, such as watching animations and completing goal setting. Key messages emerging from the online education were consistent with the strategies that participants reported using for reducing and breaking up sitting (discussed later), suggesting good coherence between education and subsequent behaviour change in this intervention. Overall, the online education was successfully delivered and led to good participant engagement. Despite this, participants had limited recall of the online education when asked at 6 months. Future interventions could, therefore, benefit from a ‘refresher’ education session to maintain continued delivery of behaviour change techniques from this component.

Health coaching has been used widely, including remote delivery, to modify lifestyle in adults with T2DM with resultant improvements in glucose control.^29^ The present study provides novel evidence of the suitability and value of health coaching for supporting changes in sedentary behaviour in this population group. The positive experiences and high engagement with the health coaching sessions as part of RESIT are reflective of sedentary behaviour interventions in other population groups. In older adults with frailty, health coaching was the most acceptable intervention component and played a central role in supporting ongoing reductions in sedentary behaviour.^30^ Like in the present intervention, encouragement and social support were central to the usefulness of the health coaching sessions.^30^ It is, therefore, recommended that health coaching is included as a core element of interventions that target sedentary behaviour reductions in clinical groups, including T2DM.

A novel element of RESIT was individual tailoring through the availability of three types of digital tools (consumer-based wearables, smartphone apps and computer software) that participants could choose from to personalise behaviour change support. The use of electronic (‘e’) and mobile (‘m’) health interventions is expanding rapidly.^31^ Evidence suggests that effects of e-health and m-health strategies can be positive, at least in the short term, but that engagement by participants with the devices and associated strategies is key to maximising success.^32^ Intervention planning should consider digital literacy skills of participants and consider ways to support this in order to optimise engagement with technological components.

In our analyses, we found that computer software that delivered prompts and reminders was less used than wearables and smartphone apps, and engagement with all three types of support declined over time. Computer software being less mobile could be one explanatory factor, especially for participants who were not office workers. Wearables had the greatest levels of engagement and both wearables and smartphone apps were perceived as very useful. In a review of smartphone apps for health behaviour change, modest effects were reported for diet, physical activity and sedentary behaviour, although apps used in conjunction with other strategies seemed more successful.^33^ These findings are supported by the present study, which demonstrated the use of a combination of strategies was successfully received by participants.

Although engagement with wearables and apps was good, and possibly better than previous studies might suggest,^32^ a small minority of participants chose not to engage in such approaches within the intervention for reasons such as app or device features not working and participants not being users of apps in general. Although not found in our data, participant burden could be an issue too. Moreover, if initial use of wearables is successful, participants may use them less over time due to improved self-regulation, habit development and increased confidence in maintaining behaviour change. Certainly, the e-health and m-health tools provided, especially wearables and smartphone apps, showed good engagement and perceived usefulness. It is, therefore, recommended that wearable and smartphone apps are used in future interventions that aim to reduce sitting in community settings.

Open-ended questionnaire and interview data confirmed that participants adopted multiple strategies to reduce and break up sitting. Breaking up sitting was an obvious strategy, such as the use of a sit-to-stand desk or standing while doing certain tasks, but participants also reported purposeful (eg, walking) and incidental (eg, short breaks) physical activity. Notably, participants did not always differentiate between sitting less and increasing physical activity, hence the demarcation between these subthemes is blurred. However, the distinction between purposeful and incidental strategies, whether active or not, might be an important one. For example, while the obvious strategy to reduce sitting is to move more, this might not be possible in some circumstances, such as when working in an office. Incidental strategies, therefore, may take on more importance and should be considered when supporting individuals in making action plans and setting goals.

Early interventions targeting reductions in sedentary behaviour sometimes focused almost exclusively on sitting less through the adoption of standing postures alone.^34^,^36^ Research now suggests that in addition to standing, more active behaviours may yield greater health benefits.^37 38^ Given the accepted ‘compositional’ model in this field, whereby movement behaviours are mutually exclusive (eg, sedentary sitting and moderate-to-vigorous physical activity cannot be undertaken simultaneously), it is reasonable and advantageous to be encouraging physical movement as one strategy to reduce sedentary sitting. Equally, an approach could be adopted that allows for graduated strategies, building from sitting to standing and then progressing to physical activity of greater intensities; this could be in the form of short movement ‘snacks’.^39^ Standing may prove to be an important behavioural transition towards greater activity. There may also be other benefits from transitioning from sitting to standing, such as mental and orthopaedic.^19 40^

The process evaluation data reported in the current paper suggests that participants used both standing and moving as viable strategies to reduce their sitting. Moreover, these could be chosen purposefully whereby a clear bout of physical activity is undertaken, or more as an incidental part of the day whereby sitting is broken up through doing another task or adopting a standing posture. Some participants mentioned inventing new or varied tasks to reduce their sitting, including doing extra cleaning in the house or running errands in less sedentary ways. Encouraging adoption of both purposeful and incidental physical activity, therefore, appears to be central for successful interventions to reduce sitting.

Work-related behaviours (eg, desk-based work; work restrictions) were clearly identified as barriers by intervention participants in the present study and are well known in the literature.^41 42^ With participants identifying work-related barriers, such as being required to work with a computer at a conventional desk, obvious solutions centre on environmental changes. This could include the provision of alternative furniture (eg, sit-to-stand desks) to enable the same work to continue while standing and opportunities to sit less during work (eg, regular breaks or other regulatory, social and environmental changes that encourage or prompt less sitting).^43 44^ Notwithstanding such potential solutions, it should be noted that some jobs may preclude taking breaks or sitting less, and some environmental solutions may be costly.

Within the home environment, social context was a key barrier to sitting less, such as conforming to sedentary postures (ie, sitting) with guests in the home. Such social constraints have rarely been considered in this field other than at work, but could be powerful influencing factors for intervention engagement.^45^ Other non-work behaviours identified as contributing to sedentary behaviour include television viewing, other leisure-time screen use and sedentary transport options,^46^ and these remain significant behavioural barriers to reducing sedentary time. The present intervention may benefit from targeted behavioural support relating to overcoming barriers in the workplace and at home.

Health and disability-related factors, as well as fatigue, also emerged as barriers to sitting less. It is important that interventions for individuals with long-term health conditions, like T2DM, recognise such barriers and work with participants to seek solutions. This can be done more effectively through adopting a co-production approach for the intervention design that actively involves the target group.^47^ With respect to addressing the barriers identified here, a prior study reported that working while standing up was useful in boosting energy and feelings of productivity at work.^13^ Increases in standing could, therefore, be explored as a strategy to overcome the barrier of perceived fatigue in individuals with T2DM. The RESIT intervention may benefit from further co-produced strategies to overcome the health-related barriers that individuals with T2DM face with reducing sitting.

In summary, this feasibility trial identified a number of important barriers to reducing sitting, but these may require greater effort to enact at scale given the commonness of office-based employment and the reinforcement value and availability of screens and car-based transport. Initiatives involving individual, social and environmental approaches are recommended in this context^44 48^ and may benefit from tailored behavioural support as provided in the present intervention.

The process evaluation also explored the potential influence of the trial’s health assessments and reasons that participants took part in the study. Perceptions of both intervention and control participants suggested that the trial’s health assessments motivated behaviour change. Behaviour change can take place from simply being observed as part of study assessments,^49^ but bias in the present study due to this factor is likely to be limited as perceptions of behaviour change from study measurements were greater in the intervention group. It is also possible that the study taking place during the COVID-19 pandemic influenced reasons for taking part, such as greater awareness of health and more time to take part.

Health motivation was clearly a factor that motivated participants to take part in the study. However, it may lead to unrepresentative sampling by not including individuals who are less motivated to improve their health. Similarly, altruistic reasons may reflect recruitment of individuals with time and interest, rather than those with the greatest needs. This is important information for future participant recruitment strategies and suggests that studies should consider participant representativeness in their samples to reflect the intended target group. These recruitment issues may need further consideration for a definitive RCT.

We conducted a comprehensive mixed-methods process evaluation that has strengthened the learnings from this feasibility trial. This will increase the chances of success of a future definitive RCT. We evaluated participants in both the intervention and control groups, in addition to interviewing the intervention’s health coaches, to explore processes related to the trial, in depth. We have also identified some limitations of this process evaluation. Despite return rates being quite good for the process evaluation questionnaires, these were not completed by all participants. It is possible that non-responders to these questionnaires may have had different perceptions related to the trial, but we were unable to test this. Moreover, open-ended responses are always prone to bias through only those willing and able to articulate responses. Regarding the generalisability of our trial, it is worth recognising the importance of language. We restricted our ‘sit less’ intervention and messages to those who were ambulatory. Future studies may wish to broaden this focus and include those who are non-ambulatory or have other movement conditions. If so, messaging around spending less time ‘still’ or ‘sedentary’, rather than ‘sitting’, may be appropriate

## Conclusion

In conclusion, our process evaluation showed that the RESIT intervention operated a largely successful online education session, delivered health coaching that had high engagement and was seen as valuable by participants, and identified wearables and smartphone apps as digital technology that is engaged with highly in a sedentary behaviour intervention. Strategies used for behaviour change were identified that are important in understanding how individuals with T2DM engage with sedentary behaviour interventions, in addition to barriers that need to be considered in future studies. These findings can be used to optimise the delivery and evaluation of the RESIT intervention in a future definitive RCT and sedentary behaviour interventions more widely, for individuals with T2DM.

## Supplementary material

10.1136/bmjopen-2025-101309 online supplemental table 1

10.1136/bmjopen-2025-101309 online supplemental figure 1

## Data Availability

Data are available in a public, open access repository.
