# Supplementary material for: Randomised-controlled feasibility study evaluating the REgulate your SItting Time (RESIT) intervention for reducing sitting in individuals with type 2 diabetes: a process evaluation
Source: BMJ Open. 2026 Feb 16;16(2):e101309. doi: 10.1136/bmjopen-2025-101309 (PMC12911826; doi:10.1136/bmjopen-2025-101309)
Supplement: online supplemental table 1 [file bmjopen-16-2-s001.docx]

**Supplementary Tables**

**Supplementary Table 1: Details of the qualitative researchers (Supporting COREQ Checklist)**

| **Initials** | **Sex** | **Task** | **Occupation & experience** | **Qualifications** |
| --- | --- | --- | --- | --- |
| MLB | F | Conduct interviews | Health promotion professional. Experience and training in multi-method research; published qualitative research. | PhD |
| EMC | F | Code and analyse interview data | Physiotherapist and health academic. Experience and training in multi-method research; published qualitative research. | PhD |
| ERH | F | Code and analyse interview data | Sport and exercise science and psychology academic. Experience and training in multi-method research; published qualitative research. | PhD |
| SJHB | M | Code and analyse open-ended survey data | Health and psychology academic. Highly cited researcher. Extensive experience of multi-method research; published qualitative research. | PhD |

Additional details:

- There were no previous relationships with participants prior to the study.
- The participants knew that the interviewer was a researcher.
- Characteristics of researchers listed in T1 above. No other information reported.
- Questions/interview guide agreed by researchers.
- No field notes taken.
- All qualitative team members agreed themes and definitions.
- Participants part of an existing RCT.
- All qualitative team members worked closely with the wider research team for topic guides, themes, definitions and write up.
- A combination of inductive and deductive approaches was utilized and themes were constructed from codes using the Framework Method to address the research question and were provided by the qualitative researchers to all other researchers.
- Main interview analyses completed by EMC and ERH; open-ended responses analysis by SJHB.
- Interviews were conducted with participant and interviewer 1:1. No one else was present.
- No repeat interviews were conducted.
- Interviews lasted approximately 60-90 minutes.
- Interview transcripts were not sent to participants.

**Supplementary Table 2. Intervention and control group process evaluation survey completion rates (N=35).**

|  | Intervention | | | | Control | | | |
| --- | --- | --- | --- | --- | --- | --- | --- | --- |
|  | At 3 months | | At 6 months | | At 3 months | | At 6 months | |
|  | N | % | N | % | N | % | N | % |
| Completed | 22 | 63 | 22 | 63 | 21 | 60 | 29 | 83 |
| Incomplete or duplicates | 6 | 17 | 8 | 23 | 3 | 9 | 3 | 9 |
| Did not attempt | 4 | 11 | 2 | 6 | 9 | 26 | 1 | 3 |
| Withdrawn from study | 3 | 9 | 3 | 9 | 2 | 6 | 2 | 6 |

% is calculated as number of responders divided by number of participants allocated to each group (N=35) multiplied by 100.

**Supplementary Table 3. Demographic characteristics of interview participants compared to all participants (at baseline)**

|  |  | Interviewed participants | | | All participants | | |
| --- | --- | --- | --- | --- | --- | --- | --- |
|  |  | Control | Intervention | All | Control | Intervention | All |
|  |  | (n = 12) | (n = 13) | (n = 25) | (n = 35) | (n = 35) | (n = 70) |
| Sex, n (%) | Male | 6 (50%) | 6 (46%) | 12 (48%) | 13 (37%) | 18 (51%) | 31 (44%) |
|  | Female | 6 (50%) | 7 (54%) | 13 (52%) | 22 (63%) | 17 (49%) | 39 (56%) |
| Age (years), mean (SD) |  | 57 (10) | 63 (11) | 60 (11) | 55 (11) | 60 (11) | 58 (11) |
| Ethnicity, n (%) | Black, Asian and minority ethnic | 10 (83%) | 6 (46%) | 16 (64%) | 22 (63%) | 21 (60%) | 43 (61%) |
|  | White (any White background) | 2 (17%) | 7 (54%) | 9 (36%) | 13 (37%) | 14 (40%) | 27 (39%) |
| Education, mean (SD) | Secondary school (e.g., high school) | 1 (8%) | 2 (15%) | 3 (12%) | 8 (23%) | 10 (29%) | 18 (26%) |
|  | Tertiary (e.g. university and above) | 11 (92%) | 11 (85%) | 22 (88%) | 27 (77%) | 25 (71%) | 52 (74%) |
| Married/cohabiting, n (%) | Married/living as married | 9 (75%) | 6 (46%) | 15 (60%) | 27 (77%) | 17 (49%) | 44 (63%) |
|  | Single/separated/divorced/widowed | 3 (25%) | 7 (54%) | 10 (40%) | 8 (23%) | 18 (51%) | 26 (37%) |
| Employment status, n (%) | Disabled | 0 (0%) | 1 (8%) | 1 (4%) | 0 (0%) | 2 (6%) | 2 (3%) |
|  | Employed full time | 7 (58%) | 3 (23%) | 10 (40%) | 23 (66%) | 12 (34%) | 35 (50%) |
|  | Employed part time | 1 (8%) | 1 (8%) | 2 (8%) | 4 (11%) | 4 (11%) | 8 (11%) |
|  | Retired | 4 (33%) | 7 (54%) | 11 (44%) | 8 (23%) | 14 (40%) | 22 (31%) |
|  | Student | 0 (0%) | 1 (8%) | 1 (4%) | 0 (0%) | 2 (6%) | 2 (3%) |
|  | Unemployed | 0 (0%) | 0 (0%) | 0 (0%) | 0 (0%) | 1 (3%) | 1 (1%) |

| **Supplementary Table 4. Online education completion rates.** |  |  |
| --- | --- | --- |
|  | N | %* |
| Did you complete the online education session? |  |  |
| Yes | 18 | 82% |
| Yes, partially | 3 | 14% |
| No | 1 | 5% |
| * % is calculated as number of responses divided by number of participants that fully completed the 3-month survey (N=22) multiplied by 100. | | |

| **Supplementary Table 5. Rates of engagement with the online education components.** | | |
| --- | --- | --- |
|  | N | %† |
| Did you complete the worksheet? |  |  |
| Yes | 18 | 86% |
| No | 3 | 14% |
|  |  |  |
| Did you read the top tips to reduce sitting time sheet? |  |  |
| Yes | 20 | 95% |
| No | 1 | 5% |
|  |  |  |
| Did you complete the goal-setting sheet? |  |  |
| Yes | 18 | 86% |
| No | 3 | 14% |
|  |  |  |
| Did you watch the animations? |  |  |
| Yes | 16 | 76% |
| No | 5 | 24% |
| † % is calculated as number of responses divided by number of participants that reported having completed (or partially completed) the online education session (N=21) multiplied by 100. | | |

| **Supplementary Table 6. Usefulness of online education session components.** | |  |
| --- | --- | --- |
|  | N | %† |
| Worksheet |  |  |
| 5 - Extremely useful | 10 | 48% |
| 4 - Very useful | 6 | 29% |
| 3 - Moderately useful | 3 | 14% |
| 2 - Somewhat useful | 0 | 0% |
| 1 - Not at all useful | 0 | 0% |
| N/A | 2 | 10% |
|  |  |  |
| Top tips |  |  |
| 5 - Extremely useful | 11 | 52% |
| 4 - Very useful | 8 | 38% |
| 3 - Moderately useful | 2 | 10% |
| 2 - Somewhat useful | 0 | 0% |
| 1 - Not at all useful | 0 | 0% |
| N/A | 0 | 0% |
|  |  |  |
| Goal setting sheet |  |  |
| 5 - Extremely useful | 8 | 38% |
| 4 - Very useful | 7 | 33% |
| 3 - Moderately useful | 3 | 14% |
| 2 - Somewhat useful | 0 | 0% |
| 1 - Not at all useful | 0 | 0% |
| N/A | 3 | 14% |
|  |  |  |
| Animations |  |  |
| 5 - Extremely useful | 10 | 48% |
| 4 - Very useful | 3 | 14% |
| 3 - Moderately useful | 5 | 24% |
| 2 - Somewhat useful | 0 | 0% |
| 1 - Not at all useful | 1 | 5% |
| N/A | 2 | 10% |
| † % is calculated as number of responses divided by number of participants that reported having completed (or partially completed) the online education session (N=21) multiplied by 100. | | |

| **Supplementary Table 7. Wearable, smartphone app(s), and computer software selections made by intervention participants at the start of the intervention (N=35).** | | |
| --- | --- | --- |
|  | N | % |
| Wearable |  |  |
| Vivofit4 (by Garmin) | 16 | 46% |
| zTrack (by MVBII) | 9 | 26% |
| Mi smart band 4 (by Xiaomi) | 7 | 20% |
| None | 1 | 3% |
| Missing data | 2 | 6% |
|  |  |  |
| Smartphone app |  |  |
| Stand up! (iPhone) | 13 | 37% |
| Sitting Timer (Android) | 8 | 23% |
| Chairless (Android) | 6 | 17% |
| None | 7 | 20% |
| Missing data | 1 | 3% |
|  |  |  |
| Computer software |  |  |
| Outstanding (Google chrome extension) | 11 | 31% |
| Break Timer app (Microsoft Windows, Apple Mac, Linux) | 9 | 26% |
| Workrave (Microsoft Windows, Linux) | 3 | 9% |
| Timeout break reminder (Apple Mac) | 2 | 6% |
| None | 9 | 26% |
| Missing data | 1 | 3% |

| **Supplementary Table 8. Rates of engagement with wearables, smartphone apps, and computer software.** | | | | |
| --- | --- | --- | --- | --- |
|  | at 3 months | | at 6 months | |
|  | N | %* | N | %‡ |
| In the last 3 months, have you used any of the apps/software/wearable devices that were suggested? |  |  |  |  |
| Yes | 19 | 86% | 16 | 73% |
| No | 3 | 14% | 6 | 27% |
|  |  |  |  |  |
| In the last 3 months, have you used any other devices/tools/methods? |  |  |  |  |
| Yes | 12 | 55% | 9 | 41% |
| No | 10 | 45% | 13 | 59% |
| * % is calculated as number of responses divided by number of participants that fully completed the 3-month survey (N=22) multiplied by 100. | | | | |
| ‡ % is calculated as number of responses divided by number of participants that fully completed the 6-month survey (N=22) multiplied by 100. | | | | |

| **Supplementary Table 9. Which wearables, smartphone apps, and computer software were used in the last 3 months?** | | | | |
| --- | --- | --- | --- | --- |
|  | at 3 months | | at 6 months | |
|  | N | %* | N | %‡ |
| Wearable used |  |  |  |  |
| Vivofit4 (by Garmin) | 8 | 36% | 6 | 27% |
| Mi smart band 4 (by Xiaomi) | 4 | 18% | 3 | 14% |
| zTrack (by MvBii) | 1 | 5% | 1 | 5% |
| zTrack (by MvBii) and my own wearable | 1 | 5% | 0 | 0% |
| Vivofit4 (by Garmin) and my own wearable | 1 | 5% | 1 | 5% |
| My own wearable | 3 | 14% | 4 | 18% |
| None of these | 4 | 18% | 7 | 32% |
|  |  |  |  |  |
| Smartphone app used |  |  |  |  |
| Stand up! (iPhone) | 5 | 23% | 5 | 23% |
| Sitting Timer (Android) | 4 | 18% | 5 | 23% |
| Chairless (Android) | 1 | 5% | 0 | 0% |
| Sitting Timer (Android) and Chairless (Android) | 1 | 5% | 0 | 0% |
| A different app | 6 | 27% | 2 | 9% |
| None of these | 5 | 23% | 10 | 45% |
|  |  |  |  |  |
| Computer software used |  |  |  |  |
| Outstanding (Google chrome extension) | 2 | 9% | 0 | 0% |
| Timeout break reminder (Computer software) | 1 | 5% | 1 | 5% |
| A different computer software | 1 | 5% | 1 | 5% |
| None of these | 18 | 82% | 20 | 91% |
| * % is calculated as number of responses divided by number of participants that fully completed the 3-month survey (N=22) multiplied by 100. | | | | |
| ‡ % is calculated as number of responses divided by number of participants that fully completed the 6-month survey (N=22) multiplied by 100. | | | | |

| **Supplementary Table 10. In the first month of the study, how often participants used a wearable, smartphone app(s), and/or computer software.** | | |
| --- | --- | --- |
|  | N | %* |
| Wearable |  |  |
| Every day | 15 | 68% |
| A few times per week | 2 | 9% |
| Once a week | 0 | 0% |
| Never | 5 | 23% |
|  |  |  |
| Smartphone app |  |  |
| Every day | 9 | 41% |
| A few times per week | 2 | 9% |
| Once a week | 2 | 9% |
| Never | 9 | 41% |
|  |  |  |
| Computer software |  |  |
| Every day | 1 | 5% |
| A few times per week | 1 | 5% |
| Once a week | 1 | 5% |
| Never | 19 | 86% |
| * % is calculated as number of responses divided by number of participants that fully completed the 3-month survey (N=22) multiplied by 100. | | |
|  | |  |

| **Supplementary Table 11. In the last three months, how often participants used a wearable, smartphone app(s), and/or computer software.** | | | | |
| --- | --- | --- | --- | --- |
|  | at 3 months | | at 6 months | |
|  | N | %* | N | %‡ |
| Wearable worn |  |  |  |  |
| 7 days/week | 14 | 64% | 11 | 50% |
| 6 days/week | 1 | 5% | 1 | 5% |
| 5 days/week | 2 | 9% | 0 | 0% |
| 4 days/week | 2 | 9% | 1 | 5% |
| 3 days/week | 0 | 0% | 1 | 5% |
| 2 days/week | 0 | 0% | 0 | 0% |
| 1 days/week | 0 | 0% | 0 | 0% |
| 0 days/week | 3 | 14% | 8 | 36% |
|  |  |  |  |  |
| Smartphone app used |  |  |  |  |
| 7 days/week | 10 | 45% | 8 | 36% |
| 6 days/week | 0 | 0% | 0 | 0% |
| 5 days/week | 1 | 5% | 1 | 5% |
| 4 days/week | 0 | 0% | 0 | 0% |
| 3 days/week | 2 | 9% | 0 | 0% |
| 2 days/week | 1 | 5% | 1 | 5% |
| 1 days/week | 1 | 5% | 0 | 0% |
| 0 days/week | 7 | 32% | 12 | 55% |
|  |  |  |  |  |
| Computer software used |  |  |  |  |
| 7 days/week | 4 | 18% | 0 | 0% |
| 6 days/week | 0 | 0% | 0 | 0% |
| 5 days/week | 1 | 5% | 0 | 0% |
| 4 days/week | 0 | 0% | 0 | 0% |
| 3 days/week | 1 | 5% | 1 | 5% |
| 2 days/week | 2 | 9% | 0 | 0% |
| 1 days/week | 0 | 0% | 0 | 0% |
| 0 days/week | 14 | 64% | 21 | 95% |
| * % is calculated as number of responses divided by number of participants that fully completed the 3-month survey (N=22) multiplied by 100. | | | | |
| ‡ % is calculated as number of responses divided by number of participants that fully completed the 6-month survey (N=22) multiplied by 100. | | | | |

| **Supplementary Table 12. Extent of agreement that the wearable, smartphone app(s), and/or computer software encouraged sitting less.** | | |  |  |
| --- | --- | --- | --- | --- |
|  | at 3 months | | at 6 months | |
|  | N | %** | N | %** |
| Wearable device has encouraged me to sit less |  |  |  |  |
| Strongly agree | 10 | 56% | 5 | 33% |
| Agree | 4 | 22% | 5 | 33% |
| Neither agree or disagree | 4 | 22% | 2 | 13% |
| Disagree | 0 | 0% | 0 | 0% |
| Strongly disagree | 0 | 0% | 3 | 20% |
| Did not use | 4 | -- | 7 | -- |
|  |  |  |  |  |
| Smartphone app has encouraged me to sit less |  |  |  |  |
| Strongly agree | 4 | 27% | 4 | 31% |
| Agree | 8 | 53% | 7 | 54% |
| Neither agree or disagree | 1 | 7% | 1 | 8% |
| Disagree | 1 | 7% | 0 | 0% |
| Strongly disagree | 1 | 7% | 1 | 8% |
| Did not use | 7 | -- | 9 | -- |
|  |  |  |  |  |
| Computer software has encouraged me to sit less |  |  |  |  |
| Strongly agree | 1 | 13% | 0 | 0% |
| Agree | 2 | 25% | 1 | 50% |
| Neither agree or disagree | 2 | 25% | 1 | 50% |
| Disagree | 1 | 13% | 0 | 0% |
| Strongly disagree | 2 | 25% | 0 | 0% |
| Did not use | 14 | -- | 20 | -- |
| ** % is calculated as number of responses divided by number of participants providing a rating for that tool at each timepoint multiplied by 100. | | | |  |

| **Supplementary Table 13. Alternate wearables, smartphone apps, and computer software participants stated using in the last 3 months.** | | | | |
| --- | --- | --- | --- | --- |
|  | at 3 months | | at 6 months | |
|  | N | %* | N | %‡ |
| Alternate wearable device used |  |  |  |  |
| Apple Watch | 1 | 5% | 1 | 5% |
| Honor 5 band | 0 | 0% | 1 | 5% |
| Mi Smart Band 6 (by Xiaomi) | 0 | 0% | 1 | 5% |
| Samsung active watch | 1 | 5% | 0 | 0% |
| Samsung galaxy fit 2 | 1 | 5% | 1 | 5% |
| Samsung watch active 2 | 1 | 5% | 0 | 0% |
| Samsung watch | 0 | 0% | 1 | 5% |
| Veryfitpro | 1 | 5% | 0 | 0% |
| No response | 17 | 77% | 17 | 77% |
|  |  |  |  |  |
| Alternate smartphone app used |  |  |  |  |
| Garmin | 1 | 5% | 0 | 0% |
| iPhone built in step counter | 1 | 5% | 0 | 0% |
| Samsung galaxy fit 2 | 1 | 5% | 0 | 0% |
| Galaxy fit | 0 |  | 1 | 5% |
| Samsung watch | 1 | 5% | 0 | 0% |
| Steps counting | 1 | 5% | 0 | 0% |
| Workbreak | 1 | 5% | 1 | 5% |
| No response | 16 | 73% | 20 | 91% |
|  |  |  |  |  |
| Alternate computer software |  |  |  |  |
| Don’t know | 1 | 5% | 0 | 0% |
| Sometimes Alexa | 0 | 0% | 1 | 5% |
| No response | 21 | 95% | 21 | 95% |
| * % is calculated as number of responses divided by number of participants that fully completed the 3-month survey (N=22) multiplied by 100. | | | | |
| ‡ % is calculated as number of responses divided by number of participants that fully completed the 6-month survey (N=22) multiplied by 100. | | | | |

| **Supplementary Table 14. In past 3 months, has anything changed in your life that has had an impact on your health-related behaviours? (Intervention participants)** | | | | | | | |
| --- | --- | --- | --- | --- | --- | --- | --- |
|  | | at 3 months | | | at 6 months | | |
|  | | N | | %* | N | | %‡ |
| Yes | | 5 | | 23% | 10 | | 45% |
| No | | 17 | | 77% | 12 | | 55% |
| * % is calculated as number of responses divided by number of participants that fully completed the 3-month survey (N=22) multiplied by 100. | | | | | | | |
| ‡ % is calculated as number of responses divided by number of participants that fully completed the 6-month survey (N=22) multiplied by 100. | | | | | | | |
| **Supplementary Table 15. In past 3 months, has anything changed in your life that has had an impact on your health-related behaviours? (Control participants)** | | | | | | | |
|  | | 3 months | | | 6 months | | |
|  | | N | %\|\| | | N | %# | |
| Yes | | 4 | 19% | | 8 | 28% | |
| No | | 17 | 81% | | 21 | 72% | |
| \|\| % is calculated as number of responses divided by number of participants that fully completed the 3-month survey (N=21) multiplied by 100. | | | | | | | |
| # % is calculated as number of responses divided by number of participants that fully completed the 6-month survey (N=29) multiplied by 100. | | | | | | | |
| **Supplementary Table 16. Has being part of the RESIT study, despite being in the control group, changed your sitting behaviour over the past 3 months? (Control participants)** | | | | | | | |
|  | at 3 months | | | at 6 months | | |  |
|  | N | %\|\| | | N | %# | |  |
| Yes | 6 | 29% | | 6 | 21% | |  |
| No | 15 | 71% | | 23 | 79% | |  |
| \|\| % is calculated as number of responses divided by number of participants that fully completed the 3-month survey (N=21) multiplied by 100. | | | | | | |  |
| # % is calculated as number of responses divided by number of participants that fully completed the 6-month survey (N=29) multiplied by 100. | | | | | | |  |

| **Supplementary Table 17. Self-reported effects of study measurements on behaviour in the intervention group.** | | | | |
| --- | --- | --- | --- | --- |
|  | at 3 months | | at 6 months | |
|  | N | %* | N | %‡ |
| The offer of study measurements encouraged me to participate in the study |  |  |  |  |
| Strongly agree | 4 | 18% | 4 | 18% |
| Agree | 11 | 50% | 9 | 41% |
| Neither agree nor disagree | 5 | 23% | 7 | 32% |
| Disagree | 2 | 9% | 0 | 0% |
| Strongly disagree | 0 | 0% | 2 | 9% |
|  |  |  |  |  |
| The measurements at the start of the study motivated me to change aspects of my behaviour |  |  |  |  |
| Strongly agree | 2 | 9% | 3 | 14% |
| Agree | 15 | 68% | 13 | 59% |
| Neither agree nor disagree | 4 | 18% | 4 | 18% |
| Disagree | 1 | 5% | 0 | 0% |
| Strongly disagree | 0 | 0% | 2 | 9% |
|  |  |  |  |  |
| The measurements at the start of the study motivated me to want to change how much time I spent sitting |  |  |  |  |
| Strongly agree | 7 | 32% | 4 | 18% |
| Agree | 11 | 50% | 14 | 64% |
| Neither agree nor disagree | 3 | 14% | 2 | 9% |
| Disagree | 1 | 5% | 0 | 0% |
| Strongly disagree | 0 | 0% | 2 | 9% |
|  |  |  |  |  |
| Knowing that follow up measurements would be taken later in the study motivated me to want to change aspects of my behaviour |  |  |  |  |
| Strongly agree | 5 | 23% | 3 | 14% |
| Agree | 10 | 45% | 15 | 68% |
| Neither agree nor disagree | 5 | 23% | 2 | 9% |
| Disagree | 2 | 9% | 0 | 0% |
| Strongly disagree | 0 | 0% | 2 | 9% |
|  |  |  |  |  |
| Knowing that follow up measurements would be taken later in the study motivated me to want to change how much time I spent sitting |  |  |  |  |
| Strongly agree | 6 | 27% | 3 | 14% |
| Agree | 10 | 45% | 14 | 64% |
| Neither agree nor disagree | 4 | 18% | 3 | 14% |
| Disagree | 2 | 9% | 0 | 0% |
| Strongly disagree | 0 | 0% | 2 | 9% |
| * % is calculated as number of responses divided by number of participants that fully completed the 3-month survey (N=22) multiplied by 100. | | | | |
| ‡ % is calculated as number of responses divided by number of participants that fully completed the 6-month survey (N=22) multiplied by 100. | | | | |

| **Supplementary Table 18. Self-reported effects of study measurements on behaviour in the control group.** | | | |  |
| --- | --- | --- | --- | --- |
|  | 3 months | | 6 months | |
|  | N | %\|\| | N | %# |
| The offer of study measurements encouraged me to participate in the study |  |  |  |  |
| Strongly agree | 0 | 0% | 2 | 7% |
| Agree | 10 | 48% | 10 | 34% |
| Neither agree nor disagree | 11 | 52% | 14 | 48% |
| Disagree | 0 | 0% | 3 | 10% |
| Strongly disagree | 0 | 0% | 0 | 0% |
|  |  |  |  |  |
| The measurements at the start of the study motivated me to change aspects of my behaviour |  |  |  |  |
| Strongly agree | 0 | 0% | 2 | 7% |
| Agree | 6 | 29% | 10 | 34% |
| Neither agree nor disagree | 12 | 57% | 11 | 38% |
| Disagree | 3 | 14% | 5 | 17% |
| Strongly disagree | 0 | 0% | 1 | 3% |
|  |  |  |  |  |
| The measurements at the start of the study motivated me to want to change how much time I spent sitting |  |  |  |  |
| Strongly agree | 0 | 0% | 2 | 7% |
| Agree | 9 | 43% | 12 | 41% |
| Neither agree nor disagree | 10 | 48% | 11 | 38% |
| Disagree | 2 | 10% | 4 | 14% |
| Strongly disagree | 0 | 0% | 0 | 0% |
|  |  |  |  |  |
| Knowing that follow up measurements would be taken later in the study motivated me to want to change aspects of my behaviour |  |  |  |  |
| Strongly agree | 0 | 0% | 1 | 3% |
| Agree | 8 | 38% | 12 | 41% |
| Neither agree nor disagree | 12 | 57% | 9 | 31% |
| Disagree | 1 | 5% | 6 | 21% |
| Strongly disagree | 0 | 0% | 1 | 3% |
|  |  |  |  |  |
| Knowing that follow up measurements would be taken later in the study motivated me to want to change how much time I spent sitting |  |  |  |  |
| Strongly agree | 0 | 0% | 1 | 3% |
| Agree | 7 | 33% | 11 | 38% |
| Neither agree nor disagree | 12 | 57% | 11 | 38% |
| Disagree | 2 | 10% | 5 | 17% |
| Strongly disagree | 0 | 0% | 1 | 3% |
| \|\| % is calculated as number of responses divided by number of participants that fully completed the 3-month survey (N=21) multiplied by 100. | | | | |
| # % is calculated as number of responses divided by number of participants that fully completed the 6-month survey (N=29) multiplied by 100. | | | | |
