# Supplementary material for: Randomised-controlled feasibility study evaluating the REgulate your SItting Time (RESIT) intervention for reducing sitting in individuals with type 2 diabetes: a process evaluation
Source: BMJ Open. 2026 Feb 16;16(2):e101309. doi: 10.1136/bmjopen-2025-101309 (PMC12911826; doi:10.1136/bmjopen-2025-101309)
Supplement: online supplemental figure 1 [file bmjopen-16-2-s002.docx]

**FULL STUDY TITLE**

A tailored intervention to reduce sitting behaviour in people with Type 2 Diabetes: A randomised-controlled feasibility study

**SHORT STUDY TITLE / ACRONYM** REgulate your SItting Time (RESIT)

**PROTOCOL VERSION NUMBER AND DATE 2 (29/04/2020)**

**RESEARCH REFERENCE NUMBERS**

| **IRAS Number:** 279157 |  |
| --- | --- |

Contents

[SIGNATURE PAGE 3](#_Toc33447948)

[KEY STUDY CONTACTS 4](#_Toc33447949)

[STUDY SUMMARY 4](#_Toc33447950)

[ROLE OF STUDY SPONSOR AND FUNDER 4](#_Toc33447951)

[ROLES AND RESPONSIBILITIES OF STUDY MANAGEMENT COMMITEES/GROUPS & INDIVIDUALS 4](#_Toc33447952)

[PROTOCOL CONTRIBUTORS 6](#_Toc33447953)

[PARTICIPANT FLOW DIAGRAM 7](#_Toc33447954)

[STUDY PROTOCOL 8](#_Toc33447955)

[BACKGROUND 8](#_Toc33447956)

[RESEARCH OBJECTIVES 10](#_Toc33447957)

[STUDY DESIGN/METHODS 10](#_Toc33447958)

[Study design 10](#_Toc33447959)

[Participants 10](#_Toc33447960)

[Sample size 10](#_Toc33447961)

[Recruitment 10](#_Toc33447962)

[Randomisation 11](#_Toc33447963)

[Intervention group 11](#_Toc33447964)

[Control group 12](#_Toc33447965)

[Data collection 13](#_Toc33447966)

[Data analysis 15](#_Toc33447967)

[DISSEMINATION OF FINDINGS 15](#_Toc33447968)

[ETHICAL AND REGULATORY COMPLIANCE 16](#_Toc33447969)

[GOOD CLIINICAL PRACTICE 16](#_Toc33447970)

[SAFETY REPORTING 16](#_Toc33447971)

[CONFIDENTIALITY AND RECORD KEEPING 17](#_Toc33447972)

# SIGNATURE PAGE

The undersigned confirm that the following protocol has been agreed and accepted and that the Chief Investigator agrees to conduct the study in compliance with the approved protocol and will adhere to the principles outlined in the Declaration of Helsinki, the Sponsor’s SOPs, and other regulatory requirement.

I agree to ensure that the confidential information contained in this document will not be used for any other purpose other than the evaluation or conduct of the investigation without the prior written consent of the Sponsor.

I also confirm that I will make the findings of the study publically available through publication or other dissemination tools without any unnecessary delay and that an honest accurate and transparent account of the study will be given; and that any discrepancies from the study as planned in this protocol will be explained.

| **Chief Investigator:** | | |
| --- | --- | --- |
| Signature: ...................................................................................................... |  | Date: ....../....../...... |
| Name: (please print):  ...................................................................................................... |  |  |

# KEY STUDY CONTACTS

| Chief Investigator | DETAILS REDACTED |
| --- | --- |
| Sponsor | Brunel University London  Kingston Lane  Uxbridge  UB8 3PH |
| Funder | Diabetes UK (grant number 19/0005972) |

# STUDY SUMMARY

| Study Title | A tailored intervention to reduce sitting behaviour in people with Type 2 Diabetes: A randomised-controlled feasibility study |
| --- | --- |
| Short title | REgulate your Sitting Time (RESIT) |
| Study Design | Randomised controlled feasibility trial |
| Study Participants | Adults with Type 2 diabetes |
| Planned Size of Sample | 70 participants |
| Planned Study Period | 18 months |

# ROLE OF STUDY SPONSOR AND FUNDER

The Sponsor of this study is the Brunel University London. The sponsor is responsible for the overall conduct and management of the study. The funder, Diabetes UK, has reviewed the study protocol as part of the grant award process. The funder will not have any involvement with the conduct of the study, data analysis or presentation of the findings.

# ROLES AND RESPONSIBILITIES OF STUDY MANAGEMENT COMMITEES

INVESTIGATOR COMMITTEE

All investigators for the project will meet every 3 months to discuss progress of the study, strategy for delivery, risk management, review of safety and efficacy data, and dissemination of findings.

TRIAL COORDINATION COMMITTEE

The trial coordination committee will comprise of the Chief Investigator, Research Assistant and Trial Coordinator and will meet fortnightly to discuss day-to-day running of the project. Updates from these meetings will be provided for the investigator committee and PSC.

PROJECT STEERING COMMITTEE (PSC)

The PSC comprises of an independent Chair, an independent expert in the study area, a lay person, the principal investigator and one co-investigator and will meet every 6 months. The PSC will advise, via the Chair, on relevant aspects of the project to the funder, sponsor and host institution. It will monitor progress of the project, protocol adherence, substantial protocol amendments and participant safety. The PSC will also advise the investigators on all aspects of the project.

PATIENT AND PUBLIC ADVISORY GROUP (PPAG)

The PPAG will implement their terms of reference from the onset which will focus on three core areas; supporting the development and direction of the research, development of information resources and dissemination of research findings. The PPAG will be comprised of the immediate project group, an independent chair, a CCG diabetes lead for the local area, a diabetes nurse and two patients with Type 2 diabetes (T2D). The PPAG will meet every 3 months and have input as below:

MANAGEMENT OF THE RESEARCH: Advisory members will help to ensure the recruitment process is practical, feasible and effective. Members will provide ideas on recruitment methods, how to encourage people with T2D to volunteer, and how to raise the subject of sitting behaviour with the target population. Members will feedback on the protocols planned for delivery within the study and will help to solve issues that may arise during delivery of the study.

DEVELOPMENT OF INFORMATION RESOURCES: Advisory members will help to make language and content of information resources appropriate and accessible to potential volunteers, including questionnaires, information leaflets and consent forms. This will aid recruitment and help potential volunteers to make informed choices about their participation.

DISSEMINATION TO THE DIABETES COMMUNITY: Advisory members will help interpret the reporting of the research and provide feedback on outputs to ensure disseminated outcomes are important to people with T2D. Members will assist with interpreting the research findings in the context of identifying themes that the researchers might miss, help check the validity of the conclusions from a diabetes patient perspective, and highlight findings that are more relevant to people with diabetes. They will also help in planning of a public outreach event to share the findings with the diabetes community and health professionals in the area.

# PROTOCOL CONTRIBUTORS

PERSONAL DETAILS REDACTED

# PARTICIPANT FLOW DIAGRAM


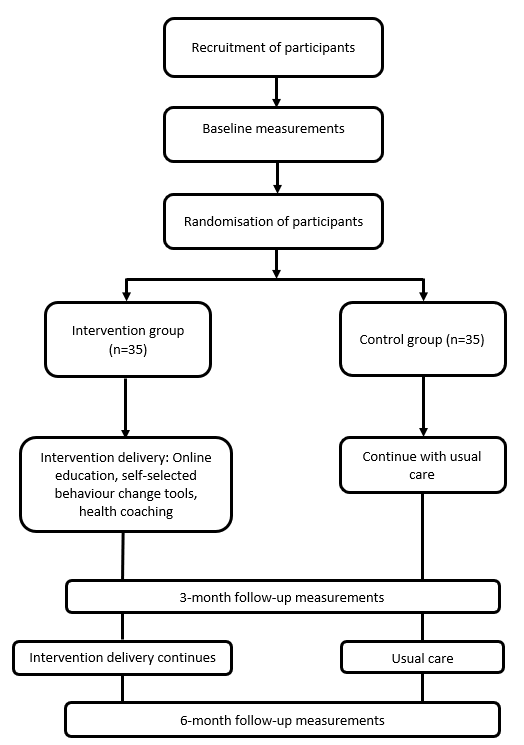


# STUDY PROTOCOL

## BACKGROUND

There are more than 3 million people with Type 2 diabetes (T2D) in the UK, costing the NHS 10% of its annual budget (1). People with T2D have a high risk of cardiovascular disease (CVD), early death, poor psychological wellbeing and a burden of diabetes-related complications such as neuropathy, nephropathy and retinopathy that reduce quality of life (2-4). Improving glycaemic control is important for reducing the onset of these secondary outcomes (5, 6) and effective interventions thus need to be identified.

People with T2D spend 8.0-9.5 hours/day sitting (7-9). Sitting (sedentary behaviour) has been detrimentally associated with CVD (10); a leading cause of death in T2D (1). Each hour of sedentary time is detrimentally associated with CVD risk markers in people with T2D including waist circumference, insulin resistance and high-density lipoprotein cholesterol (HDL) (7). Importantly, the detrimental associations of sedentary time with T2D, CVD and psychological wellbeing are independent of time spent in moderate-to-vigorous physical activity (MVPA) (10, 11). At least 60-120 minutes/day of MVPA may be needed to protect against the increased mortality risk associated with high amounts of sitting (12, 13), which is an unrealistic target for most people. In T2D, each 1 minute increase in sedentary time is related with a 0.12 minute increase in time spent in hyperglycaemia (7.4 minute increase in hyperglycaemia for each extra hour of sitting) (14). Hyperglycaemia increases CVD risk (1) and is associated with poor psychological wellbeing (15). Each hour of sedentary time is also unfavourably associated with waist circumference, insulin and a clustered CVD risk score, while each break in sedentary time is associated with lower waist circumference and improved HDL (7, 8). Reducing total and prolonged sitting is thus an important target to reduce CVD risk and poor psychological wellbeing in people with T2D.

Experimental evidence demonstrates that breaking up sitting time with 2 minute bouts of light-intensity walking every 20 minutes improves postprandial (post-meal) glucose over 5 hours in healthy adults (16-18); 5 minutes of standing or light walking every 30 minutes improved postprandial glucose and insulin in people at high risk of T2D over 7.5 hours and persisted into the following day (19); and 20 minutes of light walking every hour over 6.5 hours improved postprandial glucose, triglycerides and resting blood pressure in inactive adults (20). Breaking up sitting with light walking or light body weight resistance exercises for 3 minutes every 30 minutes acutely improved postprandial and nocturnal glucose, insulin, triglycerides and blood pressure in people with T2D (21-23). The magnitude of response in these studies is greatest in individuals with poor metabolic health, such as T2D. People with T2D should thus target reductions in sitting in addition to increases in MVPA to optimise management of their disease, which is recommended by the American Diabetes Association (24). The importance of reducing sitting time in this group is emphasised by observations that only 4-6% of UK adults meet the recommended 150 minutes/week of MVPA when measured using accelerometry (25). Interventions to reduce sitting may therefore be more achievable and better tolerated in people with T2D (26). However, there is a paucity of studies that have evaluated interventions for reducing sitting in people with T2D and strategies to achieve this in healthcare do not exist. This will be addressed by this project through the evaluation of a novel evidence-based patient-tailored self-regulation intervention based upon effective behaviour change techniques (BCTs) for reducing and breaking up sitting (27).

A number of interventions using various BCTs and self-monitoring tools have effectively reduced sitting in the general population and office workers. For example, a workplace intervention targeting reductions in sitting using a wrist-worn prompt device coupled with goal setting reduced sitting by 25 minutes/work shift over 3 days and reduced prolonged sitting (28). The use of a paper-based sitting diary as part of multicomponent workplace interventions (including providing information, goal setting, health coaching, and height-adjustable workstations) led to 50-125 minute/day reductions in workplace sitting after 4-12 weeks and 45-83 minute/day reductions after 12 months (29-31). Using computer prompt software and phone apps as part of an 8-week multicomponent workplace intervention (including providing information, goal setting and health coaching) reduced prolonged sitting by 39 minutes and increased the number of breaks from sitting by 7.8 per work shift (32). A phone app for self-monitoring sitting in addition to goal-setting and individualised feedback reduced daily sitting by 40 minutes and increased breaks from sitting by 5.7 per day after 1 week in healthy adults (33). In overweight/obese adults, after an education session on sedentary behaviour, use of a self-regulatory phone app that prompted participants to break up their sitting and provided feedback on sitting led to a 44-47 minute reduction in daily sitting after 1 week (34). Interventions incorporating BCTs such as providing information on health consequences, problem solving, goal setting, action planning, social support, restructuring the physical environment, prompts and cues, and self-monitoring thus appear to have promise for reducing sitting. However, there is a lack of literature evaluating the efficacy of reducing sitting in people with T2D. In participants with T2D, self-monitoring using a pedometer as part of a cognitive-behavioural intervention led to an increase in steps per day after 3 months and 1 year (35); while sedentary time reduced at 3 months but returned to baseline levels after 1 year; likely because the intervention did not specifically target changes in sedentary time. The lack of tested interventions in T2D will be addressed by this project.

With regards to the most promising BCTs used in previous interventions for reducing sitting, a systematic review of 38 interventions (with varying methodologies) in the general population identified that self-monitoring and problem solving were the most promising BCTs for reducing sitting, alongside restructuring the social and physical environment (27). While retrospective systematic reviews can help to identify the BCTs used in a combination of past effective versus ineffective interventions, what is needed are trials that are tailored to the needs of the population, using BCTs within one intervention that have been found to be included in effective trials, like those mentioned above. Furthermore, tailored behaviour change strategies are more likely to lead to effective outcomes, as seen when delivering interventions using Motivational Interviewing (36, 37) and those based on Self-Determination Theory (38). Thus, enabling participants to select their own tools (e.g. sitting diary, wearable tracker, phone app or computer app), while ensuring that the same BCT strategy is being used (e.g. self-monitoring of behaviour), may be a more effective approach to reducing sitting as one-size does not fit all. Although the findings of the relatively short interventions discussed above that included self-monitoring of sitting are promising, participants in these studies were not provided a choice of which self-monitoring tools they could use. One previous intervention comprised of reviewing sitting time, providing feedback on sitting and allowing participants to choose six different goals to reduce sitting, led to a 96 minute/day decrease in sitting after six weeks in older adults (39). This study shows promise but there is no research that has evaluated the effectiveness of people with T2D selecting their own tools to reduce sitting.

This study will test the more promising BCTs for reducing sitting in adults identified in previous systematic reviews (27, 40) alongside others found in effective interventions mentioned above, in a controlled tailored intervention. The intervention will allow autonomy via provision of participant-led choices of the “*delivery”* of the BCTs (e.g. via a phone app, computer app, wearable tracker and/or paper diary). However, as an intervention package, all participants will have access to the same BCTs. This is in line with a core NICE recommendation for the use of tailored care in the management of T2D (41) and the Diabetes UK research strategy target for improving self-management of diabetes.

## RESEARCH OBJECTIVES

The main objectives of this feasibility study are to:

1. Establish and refine a recruitment strategy for people with Type 2 diabetes.
2. Determine participant attrition in the trial.
3. Determine completion rates for outcome measures (and whether these are sufficiently high to provide accurate data in a full trial).
4. Assess the acceptability of randomisation to the intervention and usual care.
5. Assess the acceptability of the intervention and data collection to participants.
6. Assess intervention fidelity and adherence.
7. Explore the feasibility of collecting data (as below) to inform the design of the health economics component in a full RCT.

The secondary objectives are to derive preliminary estimates of the effect of the intervention on participants’:

1. Device measured daily sitting time, prolonged sitting time, breaks from sitting and light and moderate-to-vigorous physical activity.
2. Cardiometabolic risk markers (BMI, waist circumference, body fat%, fasting glucose, glycated haemoglobin, lipids, blood pressure)
3. Fatigue, self-efficacy for reducing sitting time, psychological wellbeing, mood, physical functioning, musculoskeletal symptoms and quality of life.

## STUDY DESIGN/METHODS

### Study design

This will be a mixed methods randomised controlled feasibility trial conducted and reported in accordance with CONSORT guidelines (42) and the TIDieR checklist (43). After baseline measures, participants will be individually randomised to one of two conditions (intervention and passive control). The 24-week intervention period will then commence and follow-up measurements will be repeated at 12 and 24 weeks after baseline measures.

This trial is likely to be conducted before society has returned to ‘normal’ after the COVID-19 pandemic. As such, contingency plans with regards to intervention delivery and data collection methods during pandemic restrictions are detailed below.

### Participants

Participants will be aged 18-85 years with physician diagnosed or self-reported T2D who are able to ambulate unassisted and self-report sitting for ≥7 h/day (this is the threshold above which mortality risk increases) (44, 45). Exclusion criteria will be using insulin medication, unable to communicate in English, pregnant, and cognitive or physical conditions interfering with the ability to stand and ambulate (with or without the use of a walking aid). Diagnosis of T2D will be confirmed from the fasting glucose measure taken at baseline. Individuals with readings below the diabetes range (7 mmol/L) will at this stage be excluded from the study.

**COVID-19 contingency:** If it is not possible to take glucose measures at baseline due to COVID-19 restrictions, participants will not be required to fast overnight prior to data collection. Participants in this case will need to self-report Type 2 diabetes diagnosis. Each participant’s GP will be notified of their participation in the study; GPs will be asked to inform the research team if their patient does not have a Type 2 diabetes diagnosis.

### Sample size

Sample sizes between 24 and 50 have been recommended for feasibility studies (46, 47). A sample size of 70 will be used in this study (n=35 in each the intervention and control groups) to allow for the different combination of intervention tools that the participants are able to select from and to allow for drop out.

### Recruitment

Participant recruitment will be via GPs where study information will be sent out via postage using Docmail and via the GP Practice SMS text messaging service (see Docmail GP invitation letter and SMS content in additional files) to potentially eligible patients using a database search. The GP invitation letter will also be provided during consultations if the GP or nurse considers their patient to be potentially eligible. All NHS recruitment is being supported by the London North West local Clinical Research Network. This is in addition to recruiting via local Diabetes UK support groups (via email send out of the Participant Information Sheet and attendance at group sessions) and social media (including a video professionally developed by Health & Care Innovations; the script for this is based on information from the PIS). A link to the video will also be accessible from the project webpage ([tinyurl.com/RESIT-information](https://tinyurl.com/RESIT-information)), which is another route that potentially eligible participants may find information about the study. Participants recruited via these latter routes will be mapped back to their GP Practice. Potentially eligible participants will be asked to express their interest by email, returning a reply slip to the research team, or by scanning a QR code provided on recruitment materials to pass their name and email address (via Qualtrics) to the research team. A researcher will then contact interested volunteers by email/telephone to screen them. Individuals who remain potentially eligible will then be invited to complete baseline data collection after providing informed consent.

### Randomisation

Eligible participants will be randomised by an independent researcher in a 1:1 (intervention:control) ratio using a fixed block size of 4. Block randomisation is used to increase the likelihood of achieving balance of numbers in the allocation to groups given the relatively small sample size, i.e. each aim is more likely to contain an equal number of individuals.

### Intervention group

The intervention will last 24 weeks and was developed based on the more promising BCTs for reducing sedentary behaviour in adults identified in a systematic review (27, 40) and based on previous effective interventions as described below. Participants will receive a standardised set of BCTs in the intervention (see Table – BCTs in intervention) but the strategies through which some BCTs are delivered will vary dependant on the optional behaviour change tool and mode of delivery selected (see Self-selected behaviour change tools below).

*This intervention is likely to be delivered and evaluated before society has returned to ‘normal’ after the COVID-19 pandemic. The proposed intervention is intended to be delivered remotely and thus requires little modification to make it appropriate for delivery during the pandemic. The online psychoeducation programme may be minimally changed to include discussion around barriers and ideas for reducing sitting under physical distancing conditions. The content of the health coaching sessions is also likely to be affected in the same way (i.e. discussion around reducing sitting in the context of pandemic restrictions) and this will be covered during training of the health coaches.*

#### Psychoeducation programme

After randomisation, all intervention participants will be provided with an online psychoeducation programme (can be accessed through a computer or via a smartphone i.e., mobile phone compatible). This programme is an adapted version of that used in our SMArT Work multicomponent intervention that led to significant and substantial reductions in workplace sitting after 12 months (48); this online psychoeducation programme is being used in the roll out of SMArT Work for free use by workplaces (https://www.smartworkandlife.co.uk/). The programme includes numerous interactive modules which will cover: 1) information on the health consequences of sitting too much (with a particular focus on diabetes), 2) benefits of reducing sitting, 3) participants reflect on their own sitting time, 4) goal setting for reducing sitting, 5) addressing potential barriers to behaviour change, And 6) the importance of self-monitoring and using prompts for behaviour change. At the end of the online programme, participants will indicate their choices with regards to the self-selected self-monitoring and prompt tools they would like to use and guidance documents for their use will be accessible from the online education programme portal.

#### Health coaching

Participants will then have a face-to-face health coaching session 1-3 days later (this will be done via video call or telephone if it cannot be done face-to-face) with a qualified health coach followed by telephone support at approximately 2, 6 and 12 weeks after baseline (it is expected participants will benefit most from more intensive support early in the intervention to help establish appropriate behaviour change strategies). A number of BCTs will be used within the health coaching sessions to include: goal setting, problem solving, action planning, review behaviour goals, discrepancy between current behaviour and goal, feedback on behaviour, self-monitoring of behaviour, social support-unspecified, information about antecedents, information about health consequences, prompts/cues, credible source, pros and cons, social reward, verbal persuasion about capability. The consultations will be semi-structured tailored sessions harnessed on the G.R.O.W (Goals, Reality, Options, Will) model (49) that will take each participant through the four stages to enhance intrinsic self-determined motivation, capability and opportunity. The initial session will focus on a discussion around Goals, Reality (barriers to reducing sitting), Options (self-selected behaviour change tools), and Will (confidence to change, how to action plan and monitor progress). Health coaches will use the content of the initial session to inform the subsequent telephone support calls. These telephone calls will involve reviewing behavioural goals, problem-solving and progress/adjustment of goals and how to achieve them. Participants will be able to contact health coaches via email/telephone outside of planned contact for additional social support if they wish (the frequency of which will be recorded). Where data can be shared between participant and health coach from the tools below, this will be used to inform health coaching sessions. Each of the health coaches will be trained by the research team to standardise the method of delivery and BCTs used during the intervention.

#### Self-selected behaviour change tools

Participants will select the behaviour change tools they would like to use for self-monitoring sitting time and prompting breaks in sitting. The use of tailored behaviour change strategies are more likely to lead to effective outcomes than fully prescribed strategies, as seen when delivering interventions using Motivational Interviewing (36, 37) and those based on Self-Determination Theory (38). In our experience with the STAND (50), SMArT Work (48), Beat the Seat (32) and other studies, we know that a one size fits all approach does not work. Thus, enabling participants to select their own tools within the same BCT strategy (e.g. self-monitoring of behaviour) may be a more effective approach to reducing sitting. Examples of self-monitoring and prompting tools can be seen in the table (Table - Overview of self-selection tools) and these were chosen as they draw from BCTs identified as being effective for reducing sitting in previous research. Each of these tools utilises strategies from the BCT taxonomy (51) and are commercially available other than the sitting diary that has been developed for this intervention. No matter what tool is chosen, the BCTs delivered from each tool, the psychoeducation programme and health coaching will combine to use the same BCTs.

These tools will be provided to participants (face-to-face or via post) or downloaded onto their phone/computer under guidance from the research assistant following completion of the online programme. Participants will be permitted to choose a maximum of one tool from each of these categories (e.g. one smartphone app, one computer prompt, one wearable device or paper-based tracker) but they are not required to choose a tool from every category. For example, a participant may choose a smartphone app and a wearable device only. The choice is theirs, thus making the intervention person-focused to increase potential effectiveness. This is consistent with Self-Determination Theory and accepted ways of boosting intrinsic motivation. The research assistant will provide a guidance document to the participant for each tool selected that will explain how to download and how it works. Participants will not receive any instructions on how often they should engage with the tools that they select given that the intervention is person-focused. The self-selected behaviour change tools used can be changed during the intervention period if a participant reports to the health coach or research team that they are not engaging with the tool. This might be due to reasons such as technical complexity, impracticality or a dislike of the tool. If there are any problems with using any of the tools during the intervention, participants will be able to contact the research assistant for troubleshooting.

### Control group

Participants randomised to this group will receive usual care. They will complete the same measurements as the intervention group. After the trial has concluded, they will be offered use of the psychoeducation programme and the self-selected behaviour change tools.

### Data collection

After eligibility screening, participants will be invited to complete baseline data collection, which will include demographic data (date of birth, sex, ethnicity, employment status, COVID-19 circumstances i.e. change to employment, social distancing or self-isolating). The below measures will be collected at baseline, 12 and 24 weeks. Participants will be required to fast for a minimum of 10 hours prior to each data collection session, which will take place at Brunel University London. At the end of each data collection session, participants will be provided with an activPAL activity monitor to wear for up to 8 days and will return this via post using a pre-paid envelope. All participants will receive a £10 amazon shopping gift voucher at each of the three data collection timepoints if they provide data and return the activity monitor to the research team.

**COVID-19 contingency:** If there are restrictions in place or advice from the government that people with Type 2 diabetes should continue to engage with physical distancing, data collection will take place remotely. Some of the measures will be removed, while some will be adapted so that the participants can perform and record these at home without the presence of a researcher. The nature of these changes or complete removal of the measure is explained for each measure proposed below. Participants will be provided with written and/or verbal guidance with regards to completion of each measure. Participants in this instance will not be required to fast overnight prior to data collection.

#### Sitting, standing and stepping

This will be measured using the activPAL device worn on the thigh for 24 hours/day for up to 8 days. The activPAL provides valid and reliable assessment of sitting, standing, stepping and postural transitions (52-56). Participants will record in a diary the time they woke up and got out of bed, time they went to bed, any times they worked, and to sleep and any periods when the device was removed. The following outcomes will be assessed: daily sitting time, time spent in prolonged sitting, the number of sit-upright transitions, standing time, stepping time, and light and MVPA steps. The device will be wrapped in a nitrile sleeve and waterproof dressing. The device is attached to the thigh using medical dressing. Participants will be asked to remove the activPAL when swimming in case it becomes detached and lost. Participants will receive a guidance document advising them on how to attach their activity monitor.

**COVID-19 contingency:** these activity monitors will be issued and returned by post, which is something we do regularly already in our research.

#### Body composition

This will be measured via body mass index (BMI), waist circumference and body fat% using bioelectrical impedance analysis. Height will be measured to the nearest 0.1 cm using a stadiometer (Seca 222, Seca Ltd, Birmingham, UK) with participants standing upright looking forwards, heels on the floor and feet together. Weight and body fat% will be measured using electronic weighing scales and bio-electrical impedance analysis (BIA) with the TANITA MC-780MA P segmental body composition analyzer (Tanita Corp., Tokyo, Japan). Participants will wear light clothing and remove shoes and socks for this measurement. This device produces valid and reliable body fat estimations in adults (57). BMI will be calculated as weight (kg) / height^2^ (cm). Waist circumference will be measured using an adjustable tape measure (Seca 201, Seca Ltd, Birmingham, UK) to the nearest 0.1 cm midway between the lowest rib and the iliac crest at the end of gentle expiration.

**COVID-19 contingency:** Participants will be asked to self-report their waist circumference at each relevant time point. They will be provided with a tape measure and written guidance on how to take this measure. Body fat %, height and weight measures will not be taken.

#### Cardiometabolic risk markers

Glycated haemoglobin will be measured using the Quo-Test HbA1c Analyzer (EKF Diagnostics, Cardiff, UK). Fasting total cholesterol, HDL, triglycerides and glucose will be measured using the CardioChek PA point-of-care-system (PTS Diagnostics, Indianapolis, Indiana). A finger prick sample technique will be used to collect capillary blood for these measures. Blood pressure will be measured on the right arm in a seated position using an Omron M5-I automatic monitoring device (Omron Matsusaka Co Ltd, Matsusaka, Japan). After 5 min of rest, the first reading will be taken with a further two readings taken with a 2 min rest between each. The average of the lowest two recordings will be used for analysis.

**COVID-19 contingency:** these measures will not be taken if it is not possible or safe to collect face-to-face data from participants.

#### Psychological, sleep, musculoskeletal and wellbeing outcomes

Perceived fatigue will be measured using the Chalder Fatigue Scale (58). Self-efficacy for reducing sitting will be assessed using an adapted version of the Schwarzer and Renner Physical Exercise Self-Efficacy Scale (59). A perceived sense of control over one’s actions and outcomes will be assessed using the Generalised Self-Efficacy Scale (60) and the Cohen Perceived Stress questionnaire will assess perceived stress (61). To assess the affective aspect of subjective wellbeing, the Positive and Negative Affect Scale will measure positive and negative mood (62, 63). The World Health Organization Five Well-Being Index will measure psychological wellbeing (64) and quality of life will be measured using the WHOQOL-BREF questionnaire (65). The Pittsburgh Sleep Quality Index questionnaire will assess sleep quality and duration (66) and musculoskeletal symptoms will be measured using the Standardised Nordic Questionnaire (67). These questionnaires will be completed online using an iPad provided to the participants following the above measures. The online questionnaires will be hosted by secure software, Qualtrics (Qualtrics, London, UK). See the questionnaires in supporting documents (Study questionnaires).

**COVID-19 contingency:** these questionnaire measures will be completed online or hard copy via post.

#### Physical function

This will be assessed using the Short Physical Performance Battery (SPPB) (68) and hand grip strength. The SPPB will be administered by trained researchers using standardised methods and includes assessment of standing balance, walking speed and rising from a chair. Standing balance will be tested using tandem, semi-tandem and side-by-side stands. For each stand type, the researcher will first demonstrate the task and will then support one arm while the participant positions their feet and then release support when the participant is ready and start timing. The timing is stopped when the feet move, the participant grasps the researcher for support or once 10 s has passed. For the semi-tandem stand, the heel of one foot (participant’s preference) is placed to the side of the first toe of the other foot. If unable to hold this position for 10 s, participants will be assessed with the feet in the side-by-side position. If the semi-tandem position is held for 10 s, participants will also be evaluated in the full tandem position with the heel of one foot directly in front of the toes of the other foot. Walking speed will be assessed using an 8-foot walking course with participants instructed to "walk to the other end of the course at your usual speed, just as if you were walking down the street to go to the store”. The walk is timed and is to be performed twice with the fastest used for analysis. For the chair stand (rising from a chair), a straight-backed chair will be placed next to a wall and participants will be instructed to fold their arms and stand up from the chair once. If they perform this successfully, the participants will be asked to stand up and sit down as quickly as possibly five times, which will be timed. Each of the SPPB assessments are scored on a 0-4 scale following published guidelines (68).

Grip strength will be measured on the dominant hand using a digital hand grip dynamometer (Takei Scientific Instruments Co.,Ltd, Yashorida, Japan) while in a standing position with the shoulder adducted and neutrally rotated and elbow in full extension. Three maximum attempts will be performed with a 1-min rest between each and the average recorded (69).

**COVID-19 contingency:** For the standing balance test, participants will be advised to do this next to a table for support in replace of the research assistant giving balance support. For walking speed, participants will be asked to identify an 8-foot clear space where they can perform the test. They will be provided with an 8-foot cardboard foldout ruler to mark out the distance. The chair sit to stand task will be performed at home if the participant has an appropriate chair that can be positioned steady against a wall. All of these tests will be completed during a Zoom video call between the participants and research assistant. The sessions will be recorded so that the research assistant can deduct the time taken to complete each test. Grip strength will not be measured.

#### Process evaluation and intervention acceptability

Process evaluation questionnaires will be completed by every participant (see Process evaluation questionnaires) and semi-structured one-to-one interviews (see Intervention and Control participant interview scripts) will be completed with intervention participants until saturation is reached. Interviews will be conducted by video call or phone if needed due to the pandemic. Interviews will aim to evaluate acceptability of the intervention including compliance with intervention components, facilitators and barriers to participation/compliance, choices made regarding the self-selected behaviour change tools, in addition to evaluating intervention fidelity. The APEASE criteria of the Behaviour Change Wheel (70) will be used to specifically uncover Affordability (Can it be delivered to budget?), Practicability (Can it be delivered as designed?), Effectiveness and cost-effectiveness (Does it work, is it cost-effective?), Acceptability (Is it judged appropriate by relevant stakeholders?), Side-effects/safety (Does it have any unwanted side-effects or unintended consequences?), and Equity (Will it reduce or increase the disparities in health/wellbeing and can it be accessed without causing disparity?). Each intervention strategy and BCT will be scored for each of the APEASE criteria (71). The research team will record completion rates for the online programme, number of face-to-face and telephone health coaching sessions, the number of times each self-selected behaviour change tool is chosen, the combinations in which they are chosen, switches in these tools made during the intervention and reasons for the switches. Interviews will also assess suitability of data collection procedures with participants (mix of control and intervention) until saturation is reached. A sample size of 13 is recommended for theory-based interview studies as this number of participants has been found to capture 97% of important codes from a total of 60 interviews (72); therefore we will aim for approximately this number. Semi-structured video call or phone interviews with all health coaches will assess the feasibility of delivering the coaching sessions (see Health coach interview script). Coaches will be asked to audio record 30% of their sessions, which will be coded using the list of BCTs included in the trial and the Motivational Interviewing Treatment Integrity scale (73) to assess fidelity. Coders will also listen out for any additional (unintended) BCTs they may have been used not in the protocol. Interviews will be recorded using Otter (which is an automatic transcription app that syncs data over an encrypted connection) in addition to an encrypted dictaphone as backup.

### Data analysis

#### Eligibility, recruitment, retention and outcome measure rates

This will include calculating eligibility ([participants eligible / participants assessed for eligibility] x 100), referral ([potentially eligible participants at recruitment sites / number of participants referred] x 100) recruitment ([participants randomised / number of eligible participants screened] x 100) and retention ([participants completing the intervention / participants enrolled] x 100) rates in addition to completion rates for the data collection measures ([participants providing full outcome data / participants completing the study] x 100). The number of participants recruited via the different recruitment strategies and reasons for ineligibility and withdrawal (including if none of the self-selected behaviour change tools are suitable to an individual’s need) will also be evaluated. Suitability of the data collection procedures will be evaluated via missing data rates and as part of the semi-structured interviews described above.

#### Process evaluation and intervention acceptability

Interviews will be recorded, transcribed verbatim and initially analysed inductively using Thematic Analysis (74), which will be facilitated using NVivo software (QRS International Pty Ltd, Victoria, Australia). This will enable identification of themes relative to APEASE, such as intervention acceptability (e.g. barriers and facilitators to engagement), appropriateness of data collection procedures and feasibility of delivering the health coaching support. Anonymous quotations (using pseudonyms) will be used from the interviews to illustrate the themes identified. Rating scales concerning the intervention will be analysed using descriptive statistics (means, SD, frequencies) and the open-ended responses will be used to identify relevant themes that may explain the quantitative responses provided.

#### Preliminary effects of the intervention

Descriptive statistics will be calculated to summarise the primary (device measured daily sitting time) and secondary outcomes (device measured prolonged sitting, breaks in sitting time, standing and stepping; body composition; cardiometabolic risk markers; psychological, sleep, musculoskeletal and wellbeing outcomes; physical function) and explore trends in the data. Continuous data will be summarised in terms of mean and standard deviation. Categorical data will be summarised in terms of frequency, counts and percentages. All baseline variables will be tabulated (no. (%), mean (SD)) by the treatment allocation and overall. Statistical analyses will be performed using SPSS v22 (IBM, Armonk, NY, USA).

# DISSEMINATION OF FINDINGS

Findings will be disseminated to lay, academic, practice, and policy-based audiences including:

- Summary of findings to participants via a newsletter.
- Dedicated University webpage, newsletters and social media.
- Summary report to key stakeholders in our networks e.g. Clinical commissioning group, GP practices, Diabetes UK Support Groups, Leicester Diabetes Centre.
- Publication in an international peer-reviewed journal e.g. Diabetologia.
- Presentation at conferences (e.g. International Society for Physical Activity and Health Congress).
- The National Institute for Health Research’s Collaborations for Leadership in Applied Health Research and Care, the NHS Academic Health Science Network, and the NIHR Leicester Biomedical Research Centre.

# ETHICAL AND REGULATORY COMPLIANCE

Before the start of the study, a favourable opinion will be sought from the Brunel University London Research Ethics Committee and the NHS Research Ethics Committee.

- Substantial amendments that require review by NHS REC will not be implemented until that review is in place and other mechanisms are in place to implement at site.
- All correspondence with the REC will be retained.
- It is the Chief Investigator’s responsibility to produce the annual reports as required. The Chief Investigator will notify the REC of the end of the study.
- An annual progress report (APR) will be submitted to the REC within 30 days of the anniversary date on which the favourable opinion was given, and annually until the study is declared ended.
- If the study is ended prematurely, the Chief Investigator will notify the REC, including the reasons for the premature termination.
- Within one year after the end of the study, the Chief Investigator will submit a final report with the results, including any publications/abstracts, to the REC.

The study will conform to the Declaration of Helsinki.

## GOOD CLIINICAL PRACTICE

The Chief Investigator will ensure that the study is conducted in line with the ICH Guideline for good clinical practice.

## SAFETY REPORTING

Adverse Events (AEs) are any unfavourable and unintended signs, including abnormal laboratory results, symptoms or a disease associated with treatment. If an AE occurs, this must be reported on a Case Report Form with the following information: description, date of onset and end date, severity, assessment of relatedness to study, and action taken. AEs considered related to the study as judged by the Chief Investigator will be followed until resolution or the event is considered stable. All related AEs that result in a participant’s withdrawal from the study or are present at the end of the study, should be followed up until a satisfactory resolution occurs.

Serious Adverse Events (SAEs) are defined as any untoward medical occurrence(s) that at any dose results in death, hospitalisation or prolongation of existing hospitalisation, persistent or significant disability/incapacity or a congenital anomaly or birth defect.

Suspected Serious Adverse reactions (SSARs) are any ARs considered consistent with information available about an Investigational medicinal Product (IMP).

As this study involves only a lifestyle modification, no SAEs or SSARs are expected.

## CONFIDENTIALITY AND RECORD KEEPING

All participant information documentation will be stored and protected to comply with GDPR guidance. Any paperwork relating to research activities and participant information will be stored in a locked filing cabinet at Brunel University London. Information stored on computers will be protected by passwords. Only members of the research team will have access to the data. Prior to testing, participants will be assigned a ‘participant ID number’ allocated to them by the research team. Participants will be allocated this number in a chronological order upon entry (consent) to the study. This number will be used on all data collection sheets and/or spreadsheets instead of a participant name to maintain anonymity. The data will be stored in linked fashion to enable matching of data for each participant at the different data collection time points.

Audio recorded discussions for purpose of the interviews will be recorded using Otter (<https://otter.ai/login>) and an encrypted Dictaphone (so that there is a backup). Otter is an automatic transcription app that syncs data over an encrypted connection and is stored in a secure data centre that has physical and electronic security. Participant data will only be accessible to the research team. When the user deletes files from the Otter app, they are permanently deleted from the app’s server. The research team will have a password protected account with the app that is only accessible to the Chief Investigator and members of the team involved with data analysis. Recordings on the Otter app will be downloaded onto a password protected computer as soon as possible after each interview and then deleted from the app. The encrypted Dictaphone will be stored in a locked filing cabinet at Brunel University London and will only be transported for the purpose of interviews. The recording will be saved onto a password protected computer as soon as possible after each interview and then deleted from the Dictaphone. Pseudonyms will be used in the transcribed interviews to maintain anonymity. Zoom calls will be recorded and saved directly onto a password protected university laptop or computer. Audio and video recordings will be stored for a maximum of 5 years after completion of the research and will then be deleted.

**REFERENCES**

1. Diabetes UK. Diabetes: Facts and Stats2015 Accessed 23 March 2016. Available from: <https://www.diabetes.org.uk/Documents/Position%20statements/Diabetes%20UK%20Facts%20and%20Stats_Dec%202015.pdf>

2. International Diabetes Federation. IDF Diabetes Atlas, 8th ed2017 Accessed 11 July 2018. Available from: <http://diabetesatlas.org/resources/2017-atlas.html>.

3. Ali S, Stone MA, Peters JL, Davies MJ, Khunti K. The prevalence of co-morbid depression in adults with Type 2 diabetes: a systematic review and meta-analysis. Diabet Med. 2006;23(11):1165-73.

4. Lloyd A, Sawyer W, Hopkinson P. Impact of long-term complications on quality of life in patients with type 2 diabetes not using insulin. Value Health. 2001;4(5):392-400.

5. Van der Does FE, De Neeling JN, Snoek FJ, Kostense PJ, Grootenhuis PA, Bouter LM, et al. Symptoms and well-being in relation to glycemic control in type II diabetes. Diabetes Care. 1996;19(3):204-10.

6. Borg R, Kuenen JC, Carstensen B, Zheng H, Nathan DM, Heine RJ, et al. HbA(1)(c) and mean blood glucose show stronger associations with cardiovascular disease risk factors than do postprandial glycaemia or glucose variability in persons with diabetes: the A1C-Derived Average Glucose (ADAG) study. Diabetologia. 2011;54(1):69-72.

7. Cooper AR, Sebire S, Montgomery AA, Peters TJ, Sharp DJ, Jackson N, et al. Sedentary time, breaks in sedentary time and metabolic variables in people with newly diagnosed type 2 diabetes. Diabetologia. 2012;55(3):589-99.

8. Cooper AJ, Brage S, Ekelund U, Wareham NJ, Griffin SJ, Simmons RK. Association between objectively assessed sedentary time and physical activity with metabolic risk factors among people with recently diagnosed type 2 diabetes. Diabetologia. 2014;57(1):73-82.

9. Loprinzi PD. Accelerometer-determined sedentary and physical activity estimates among older adults with diabetes: considerations by demographic and comorbidity characteristics. J Aging Phys Act. 2014;22(3):432-40.

10. Wilmot EG, Edwardson CL, Achana FA, Davies MJ, Gorely T, Gray LJ, et al. Sedentary time in adults and the association with diabetes, cardiovascular disease and death: systematic review and meta-analysis. Diabetologia. 2012;55(11):2895-905.

11. Hamer M, Coombs N, Stamatakis E. Associations between objectively assessed and self-reported sedentary time with mental health in adults: an analysis of data from the Health Survey for England. BMJ Open. 2014;4(3):e004580.

12. Ekelund U, Steene-Johannessen J, Brown WJ, Fagerland MW, Owen N, Powell KE, et al. Does physical activity attenuate, or even eliminate, the detrimental association of sitting time with mortality? A harmonised meta-analysis of data from more than 1 million men and women. Lancet. 2016;388(10051):1302-10.

13. Pulsford RM, Stamatakis E, Britton AR, Brunner EJ, Hillsdon M. Associations of sitting behaviours with all-cause mortality over a 16-year follow-up: the Whitehall II study. Int J Epidemiol. 2015;44(6):1909-16.

14. Fritschi C, Park H, Richardson A, Park C, Collins EG, Mermelstein R, et al. Association Between Daily Time Spent in Sedentary Behavior and Duration of Hyperglycemia in Type 2 Diabetes. Biol Res Nurs. 2016;18(2):160-6.

15. Zhai L, Zhang Y, Zhang D. Sedentary behaviour and the risk of depression: a meta-analysis. Br J Sports Med. 2015;49(11):705-9.

16. Bailey DP, Broom DR, Chrismas BC, Taylor L, Flynn E, Hough J. Breaking up prolonged sitting time with walking does not affect appetite or gut hormone concentrations but does induce an energy deficit and suppresses postprandial glycaemia in sedentary adults. Appl Physiol Nutr Metab. 2016;41(3):324-31.

17. Bailey DP, Locke CD. Breaking up prolonged sitting with light-intensity walking improves postprandial glycemia, but breaking up sitting with standing does not. J Sci Med Sport. 2015;18(3):294-8.

18. Dunstan DW, Kingwell BA, Larsen R, Healy GN, Cerin E, Hamilton MT, et al. Breaking up prolonged sitting reduces postprandial glucose and insulin responses. Diabetes Care. 2012;35(5):976-83.

19. Henson J, Davies MJ, Bodicoat DH, Edwardson CL, Gill JM, Stensel DJ, et al. Breaking Up Prolonged Sitting With Standing or Walking Attenuates the Postprandial Metabolic Response in Postmenopausal Women: A Randomized Acute Study. Diabetes Care. 2016;39(1):130-8.

20. Champion RB, Smith LR, Smith J, Hirlav B, Maylor BD, White SL, et al. Reducing prolonged sedentary time using a treadmill desk acutely improves cardiometabolic risk markers in male and female adults. J Sports Sci. 2018;36(21):2484-91.

21. Dempsey PC, Blankenship JM, Larsen RN, Sacre JW, Sethi P, Straznicky NE, et al. Interrupting prolonged sitting in type 2 diabetes: nocturnal persistence of improved glycaemic control. Diabetologia. 2017;60(3):499-507.

22. Dempsey PC, Larsen RN, Sethi P, Sacre JW, Straznicky NE, Cohen ND, et al. Benefits for Type 2 Diabetes of Interrupting Prolonged Sitting With Brief Bouts of Light Walking or Simple Resistance Activities. Diabetes Care. 2016;39(6):964-72.

23. Dempsey PC, Sacre JW, Larsen RN, Straznicky NE, Sethi P, Cohen ND, et al. Interrupting prolonged sitting with brief bouts of light walking or simple resistance activities reduces resting blood pressure and plasma noradrenaline in type 2 diabetes. J Hypertens. 2016;34(12):2376-82.

24. Colberg SR, Sigal RJ, Yardley JE, Riddell MC, Dunstan DW, Dempsey PC, et al. Physical Activity/Exercise and Diabetes: A Position Statement of the American Diabetes Association. Diabetes Care. 2016;39(11):2065-79.

25. National Health Service. Health Survey for England—2008: physical activity and fitness 2009 Accessed 6 February 2016. Available from: <http://www.hscic.gov.uk/catalogue/PUB00430/heal-surv-phys-acti-fitn-eng-2008-rep-v2.pdf>.

26. Henson J, Dunstan DW, Davies MJ, Yates T. Sedentary behaviour as a new behavioural target in the prevention and treatment of type 2 diabetes. Diabetes Metab Res Rev. 2016;32 Suppl 1:213-20.

27. Gardner B, Smith L, Lorencatto F, Hamer M, Biddle SJ. How to reduce sitting time? A review of behaviour change strategies used in sedentary behaviour reduction interventions among adults. Health Psychol Rev. 2016;10(1):89-112.

28. Swartz AM, Rote AE, Welch WA, Maeda H, Hart TL, Cho YI, et al. Prompts to disrupt sitting time and increase physical activity at work, 2011-2012. Prev Chronic Dis. 2014;11:E73.

29. Healy GN, Eakin EG, Owen N, Lamontagne AD, Moodie M, Winkler EA, et al. A Cluster Randomized Controlled Trial to Reduce Office Workers' Sitting Time: Effect on Activity Outcomes. Med Sci Sports Exerc. 2016;48(9):1787-97.

30. Healy GN, Eakin EG, Lamontagne AD, Owen N, Winkler EA, Wiesner G, et al. Reducing sitting time in office workers: short-term efficacy of a multicomponent intervention. Prev Med. 2013;57(1):43-8.

31. Neuhaus M, Healy GN, Dunstan DW, Owen N, Eakin EG. Workplace sitting and height-adjustable workstations: a randomized controlled trial. Am J Prev Med. 2014;46(1):30-40.

32. Maylor BD, Edwardson CL, Zakrzewski-Fruer JK, Champion RB, Bailey DP. Efficacy of a Multicomponent Intervention to Reduce Workplace Sitting Time in Office Workers: A Cluster Randomized Controlled Trial. J Occup Environ Med. 2018;60(9):787-95.

33. Arrogi A, Bogaerts A, Seghers J, Devloo K, Vanden Abeele V, Geurts L, et al. Evaluation of stAPP: a smartphone-based intervention to reduce prolonged sitting among Belgian adults. Health Promot Int. 2019;34(1):16-27.

34. Bond DS, Thomas JG, Raynor HA, Moon J, Sieling J, Trautvetter J, et al. B-MOBILE--a smartphone-based intervention to reduce sedentary time in overweight/obese individuals: a within-subjects experimental trial. PLoS One. 2014;9(6):e100821.

35. De Greef K, Deforche B, Tudor-Locke C, De Bourdeaudhuij I. A cognitive-behavioural pedometer-based group intervention on physical activity and sedentary behaviour in individuals with type 2 diabetes. Health Educ Res. 2010;25(5):724-36.

36. Rubak S, Sandbaek A, Lauritzen T, Christensen B. Motivational interviewing: a systematic review and meta-analysis. Br J Gen Pract. 2005;55(513):305-12.

37. Chen SM, Creedy D, Lin HS, Wollin J. Effects of motivational interviewing intervention on self-management, psychological and glycemic outcomes in type 2 diabetes: a randomized controlled trial. Int J Nurs Stud. 2012;49(6):637-44.

38. Silva MN, Vieira PN, Coutinho SR, Minderico CS, Matos MG, Sardinha LB, et al. Using self-determination theory to promote physical activity and weight control: a randomized controlled trial in women. J Behav Med. 2010;33(2):110-22.

39. Lewis LK, Rowlands AV, Gardiner PA, Standage M, English C, Olds T. Small Steps: Preliminary effectiveness and feasibility of an incremental goal-setting intervention to reduce sitting time in older adults. Maturitas. 2016;85:64-70.

40. Brierley ML, Chater AM, Smith LR, Bailey DP. The Effectiveness of Sedentary Behaviour Reduction Workplace Interventions on Cardiometabolic Risk Markers: A Systematic Review. Sports Med. 2019;49(11):1739-67.

41. National Institute for Health and Care Excellence. Type 2 diabetes in adults: management. Available at: <https://www.nice.org.uk/guidance/ng28/resources/type-2-diabetes-in-adults-management-1837338615493> (accessed 12 Sept 2016). 2015.

42. Eldridge SM, Chan CL, Campbell MJ, Bond CM, Hopewell S, Thabane L, et al. CONSORT 2010 statement: extension to randomised pilot and feasibility trials. BMJ. 2016;355:i5239.

43. Hoffmann TC, Glasziou PP, Boutron I, Milne R, Perera R, Moher D, et al. Better reporting of interventions: template for intervention description and replication (TIDieR) checklist and guide. BMJ : British Medical Journal. 2014;348:g1687.

44. Chau JY, Grunseit AC, Chey T, Stamatakis E, Brown WJ, Matthews CE, et al. Daily sitting time and all-cause mortality: a meta-analysis. PLoS One. 2013;8(11):e80000.

45. Ku PW, Steptoe A, Liao Y, Hsueh MC, Chen LJ. A cut-off of daily sedentary time and all-cause mortality in adults: a meta-regression analysis involving more than 1 million participants. BMC Med. 2018;16(1):74.

46. Sim J, Lewis M. The size of a pilot study for a clinical trial should be calculated in relation to considerations of precision and efficiency. J Clin Epidemiol. 2012;65(3):301-8.

47. Julious SA. Sample size of 12 per group rule of thumb for a pilot study. Pharm Stat. 2005;4(4):287-91.

48. Edwardson CL, Yates T, Biddle SJH, Davies MJ, Dunstan DW, Esliger DW, et al. Effectiveness of the Stand More AT (SMArT) Work intervention: cluster randomised controlled trial. BMJ. 2018;363:k3870.

49. Whitmore J. Coaching for Performance: GROWing People, Performance and Purpose. London: Nicholas Brealey Publishing; 2002.

50. Biddle SJ, Edwardson CL, Wilmot EG, Yates T, Gorely T, Bodicoat DH, et al. A Randomised Controlled Trial to Reduce Sedentary Time in Young Adults at Risk of Type 2 Diabetes Mellitus: Project STAND (Sedentary Time ANd Diabetes). PLoS One. 2015;10(12):e0143398.

51. Michie S, Richardson M, Johnston M, Abraham C, Francis J, Hardeman W, et al. The behavior change technique taxonomy (v1) of 93 hierarchically clustered techniques: building an international consensus for the reporting of behavior change interventions. Ann Behav Med. 2013;46(1):81-95.

52. Lyden K, Kozey Keadle SL, Staudenmayer JW, Freedson PS. Validity of two wearable monitors to estimate breaks from sedentary time. Med Sci Sports Exerc. 2012;44(11):2243-52.

53. Grant PM, Ryan CG, Tigbe WW, Granat MH. The validation of a novel activity monitor in the measurement of posture and motion during everyday activities. Br J Sports Med. 2006;40(12):992-7.

54. Ryan CG, Grant PM, Tigbe WW, Granat MH. The validity and reliability of a novel activity monitor as a measure of walking. Br J Sports Med. 2006;40(9):779-84.

55. Hart TL, Ainsworth BE, Tudor-Locke C. Objective and subjective measures of sedentary behavior and physical activity. Med Sci Sports Exerc. 2011;43(3):449-56.

56. Edwardson CL, Rowlands AV, Bunnewell S, Sanders J, Esliger DW, Gorely T, et al. Accuracy of Posture Allocation Algorithms for Thigh- and Waist-Worn Accelerometers. Med Sci Sports Exerc. 2016;48(6):1085-90.

57. von Hurst P, Walsh D, Conlon C, Ingram M, Kruger R, Stonehouse W. Validity and reliability of bioelectrical impedance analysis to estimate body fat percentage against air displacement plethysmography and dual-energy X-ray absorptiometry. Nutr Diet. 2016;73:197-204.

58. Chalder T, Berelowitz G, Pawlikowska T, Watts L, Wessely S, Wright D, et al. Development of a fatigue scale. J Psychosom Res. 1993;37(2):147-53.

59. Schwarzer R, Renner B. Social-cognitive predictors of health behavior: action self-efficacy and coping self-efficacy. Health Psychol. 2000;19(5):487-95.

60. Schwarzer R, Jerusalem M. Generalized Self-efficacy Scale. In: J. Weinman SW, & M. Johnston, editor. Measures in Health Psychology: A User’s Portfolio Causal and Control Beliefs. Windsor, UK: Nfer-Nelson; 1995. p. 35-7.

61. Cohen S, Kamarck T, Mermelstein R. A global measure of perceived stress. J Health Soc Behav. 1983;24(4):385-96.

62. Watson D, Clark LA, Tellegen A. Development and validation of brief measures of positive and negative affect: the PANAS scales. J Pers Soc Psychol. 1988;54(6):1063-70.

63. Crawford JR, Henry JD. The positive and negative affect schedule (PANAS): construct validity, measurement properties and normative data in a large non-clinical sample. Br J Clin Psychol. 2004;43(Pt 3):245-65.

64. Bech P. WHO (five) well-being index (1998 version). <https://www.psykiatri-regionh.dk/who-5/Documents/WHO5_English.pdf>. 1998.

65. World Health Organization. WHOQOL-BREF introduction, administration, scoring and generic version of the assessment: Field Trial Version. <http://www.who.int/mental_health/media/en/76.pdf>. Accessed 28 September 2016. 1996.

66. Buysse DJ, Reynolds CF, 3rd, Monk TH, Berman SR, Kupfer DJ. The Pittsburgh Sleep Quality Index: a new instrument for psychiatric practice and research. Psychiatry Res. 1989;28(2):193-213.

67. Kuorinka I, Jonsson B, Kilbom A, Vinterberg H, Biering-Sorensen F, Andersson G, et al. Standardised Nordic questionnaires for the analysis of musculoskeletal symptoms. Appl Ergon. 1987;18(3):233-7.

68. Guralnik JM, Simonsick EM, Ferrucci L, Glynn RJ, Berkman LF, Blazer DG, et al. A short physical performance battery assessing lower extremity function: association with self-reported disability and prediction of mortality and nursing home admission. J Gerontol. 1994;49(2):M85-94.

69. Incel N, Ceceli E, Durukan P, Erdem H, Yorgancioglu Z. Grip Strength: Effect of Hand Dominance. Singapore Med J. 2002;43(5):234-7.

70. Michie S, Atkins L, West R. The Behaviour Change Wheel: A Guide to Designing Interventions. London: Silverback Publishing; 2014.

71. Public Health England. Achieving behaviour change: A guide for local government and partners. 2019.

72. Francis JJ, Johnston M, Robertson C, Glidewell L, Entwistle V, Eccles MP, et al. What is an adequate sample size? Operationalising data saturation for theory-based interview studies. Psychol Health. 2010;25(10):1229-45.

73. Moyers TB, Rowell LN, Manuel JK, Ernst D, Houck JM. The Motivational Interviewing Treatment Integrity Code (MITI 4): Rationale, Preliminary Reliability and Validity. J Subst Abuse Treat. 2016;65:36-42.

74. Braun V, Clarke V. Using thematic analysis in psychology. Qualitative Research in Psychology. 2006;3(2):77-101.
