# Supplementary material for: Randomised-controlled feasibility study evaluating the REgulate your SItting Time (RESIT) intervention for reducing sitting in individuals with type 2 diabetes: a process evaluation
Source: BMJ Open. 2026 Feb 16;16(2):e101309. doi: 10.1136/bmjopen-2025-101309 (PMC12911826; doi:10.1136/bmjopen-2025-101309)
Supplement: Supplementary file 3 [file bmjopen-2025-101309.reviewer_comments.pdf]

PEER REVIEW HISTORY

BMJ Open publishes all reviews undertaken for accepted manuscripts. Reviewers are asked to complete a checklist review form and are provided with free text boxes to elaborate on their assessment. These free text comments are reproduced below.

ARTICLE DETAILS

Title (Provisional)

Process evaluation of a randomised-controlled feasibility study evaluating the REgulate your SItting Time (RESIT) intervention for reducing sitting in individuals with type 2 diabetes

Authors

Biddle, Stuart J.H.; Brierley, Marsha L; Castle, Ellen; Hunt, Emily R; Chater, Angel; Edwardson, Charlotte; Bailey, Daniel

---

VERSION 1 - REVIEW

**Reviewer**	**1**
**Name**	**Carter, Sophie E**
**Affiliation**	**York St John University**
**Date**	**08-Apr-2025**
**COI**	**None**

Thank you for the invitation to review this manuscript. The manuscript is well written and provides a detailed process evaluation of the RESIT intervention for adults with type 2 diabetes. Acceptability and feasibility findings from this trial have been previously reported, therefore, this paper provides further evaluation of the intervention, which will collectively allow for more research-informed interventions to be developed and assessed. Please see below specific comments that would further improve the manuscript:

Abstract

1. Results: When reporting the results, for clarity it would be beneficial if it was clear which data represents data from participants and which (if any) is from the health coaches. Both are documented as being involved in the 'outcome measures' section.

2. Line 28-29: Appreciate there are word count restrictions, but it would be beneficial to make it clearer what 'study evaluation methods' were that could have influenced behaviours, as these are not documented in preceding sections of the abstract.

Introduction:

3. Lines 48-50: It would be beneficial to make clear that sitting is a form of sedentary behaviour. The first sentence describes sedentary behaviours, then the next describes breaking up sitting, but how these behaviours are linked is not made clear

4. Line 69: It would be more appropriate and representative of the aim of the intervention if this was described as ‘break up periods of sitting’ rather than ‘inactivity’

5. Lines 70-72: It would be beneficial to make it clear here who specifically found the interview to be feasible and acceptable i.e., was it participants and health coaches?

Methods

6. General: As this later emerges in interview data, it would be valuable to describe when the study was conducted (i.e., dates) and when the intervention took place making reference to the health landscape at this time i.e., it was during a global pandemic

7. Line 98: Please rephrase to ‘were involved with the delivery of’

8. Questionnaires: Can the authors provide any details regarding how the items of the questionnaire were developed? It is noted that in the paper cited as reference number 15, the methods describes that ‘A process evaluation questionnaire that was developed following Medical Research Council guidelines [35] and previous research [36]’ – if this describes what is reported in this study, this detail should be included

9. Interviews: Please describe where these were conducted i.e., the environment for in-person interviews or if online

10. Interviews: Was the interviewer (MLB) involved in any other stages of the project delivery or engagement with participants with the intervention? It would be important to make this clear to understand if participant’s responses could be biased by their involvement in the intervention or not

11. Line 129: Can the sex characteristics and mean age of the health coaches be included, as has been reported for the intervention and control group participants

12. Lines 147-148: Please consider reviewing the phrase ‘NVivo for analysis’ since this supports researchers to organise, code, and interpret qualitative data, but the analysis itself is conducted by the researcher not the software

13. Line 157: A reference is needed where (ref) is currently written

14. Patient and Public Involvement: Did the researchers work with the PPI group in any way to develop the interview schedule? Were any pilots of the interview schedule or survey conducted to ensure comprehension?

Results

15. Lines 172: It would be beneficial to make it clear here at the start of this section if themes are derived from interviews with participants and/or health coaches.

16. Line 185: It would be beneficial to make it clear here this represents interview data with the participants (i.e., not also the health coaches)

17. Reasons for participation: It would be beneficial to make it clearer specifically what other sub-themes were. One is named as ‘health motivation’ but others are not explicitly stated. For example, it is assumed ‘additional time’ and ‘altruism’ are other sub-themes?

18. Line 210: For clarity, it would be beneficial to specify this data is from the questionnaires.

19. Line 220: Please revise this statement to align with terminology used in Supplementary Table 4. The rows in the table describe ‘5 extremely useful, 4 very useful, 3 moderately useful, and 2 somewhat useful’. In the text, it describes ‘useful or extremely useful. Does ‘useful’ incorporate all statements 2 – 4? Furthermore, to support this statement in the manuscript itself, could the authors provide a summary/mean/range of percentage values to quantify what they mean by ‘nearly all’.

20. Lines 230-232: Is this from data that has been reported previously? If so, it should be made clear

21. Lines 264: Please make it clear if when the intervention group ‘were asked’ if this is via interview or questionnaire data collection methods

22. Lines 384-397: This sentence feels more of a discussion of the findings, rather than reporting of results

23. Lines 395: Please make it clear if when the intervention group ‘were asked’ if this is via interview or questionnaire data collection methods

Discussion

24. Line 464: Has health coaching always been delivered remotely or has this also been via in-person sessions? This could be an important discussion point if similar benefits/engagement have been reported by participants, despite the remote nature of delivery

25. Lines 477-478: Do the authors have a reference to support this statement regarding the rapid expansion of e- and m-health interventions? Also, whilst it may seem obvious, for clarity, it would be beneficial to explain why there is an ‘inevitable’ increase in the use of these types of intervention strategy

26. Lines 484-485: Can the authors offer any thoughts on why the computer software was less adopted? Is this in line with any other research assessing computer software interventions to reduce sitting time, or research comparing computer software interventions and wearables?

27. Lines 489-490: It would be beneficial to align this point to the current intervention, i.e., RESIT used a combination of strategies and has been successfully received by participants

28. Lines 537-539: It is important to note with this statement that whilst environmental changes may be ‘obvious’ they may not always be possible in certain work environments. For example, the cost of alternative furniture may not be feasible for certain workplaces. Furthermore, permitting high frequency breaks may not be permissible in environments such as contact centres or customer-facing roles.

29. Line 565: It would be important to mention in this section regarding reasons for taking part that the COVID-19 pandemic, when this study was completed, may have also had an impact. In the results section participants reported having more time to take part due to COVID-19 and working from home/having more time– which may be less likely in a future implementation of the intervention as restrictions have now eased. Furthermore, could participant’s awareness of the need to contribute to health research have been heightened due to the pandemic and associated news stories around health/health-related research?

**Reviewer**	**2**
**Name**	**Airlie, Jennifer**
**Affiliation**	**Durham University, Department of Sport and Exercise Sciences**
**Date**	**24-Oct-2025**
**COI**	**None**

Comments for the author:

Thank you for the opportunity to review this manuscript which reports on the findings from a mixed methods process evaluation conducted within randomised-controlled feasibility study evaluating the REgulate your SItting Time (RESIT) intervention for reducing sitting in individuals with type 2 diabetes. On reviewing the manuscript, I referred to the MRC process evaluation guidance (Moore et al, 2015); hence many of my comments are as a direct result of utilising this.

Overall, the manuscript makes a useful contribution. the authors explain the rationale for undertaking a process evaluation alongside a feasibility trial well and its link to main feasibility trial is clear. Further, the data collection methods chosen for the evaluation are appropriate and the authors describe how the data was analysed well. The results are generally interpreted in line with existing evidence and reported clearly. Within the discussion the authors explore the potential mechanisms of impact of the intervention and start to discuss recommendations and implications for future work. It is refreshing that the authors acknowledged the potential influence of the assessments conducted as part of the trial

However, I feel several important reporting issues should be addressed prior to publication:

Major points:

1. I note that there that the interventions theory of change is not reported on therefore no rationale for the focus of the research questions being addressed is provided. Given the theory of change should guide which mechanisms and processes are examined, I recommend the authors include a succinct description (and ideally a figure or logic model) that sets out the hypothesised causal pathways and the specific mechanisms the evaluation sought to test.

2. Although a well-written aim is provided, on reading the manuscript in its entirety I feel it does not fully deliver on this aim as not all of the findings from the process evaluation are reported on. Whilst the results section transparently states that other findings are reported elsewhere and cites that work, I feel this manuscript would benefit from a brief justification for reporting findings across separate papers. I also wonder if the aim should be re-written to reflect the narrower scope of this manuscript?

3. I feel the authors need to provide a clear rationale for interviewing only a subset of participants (including how the subset was selected) and provide comment on whether those interviewed were representative of the trial sample. I would also ask that the authors include details on the sampling strategy (e.g., purposive for variation, convenience, or pragmatic) and any demographic differences between interviewees and non-interviewees that might influence interpretation.

4. Although questionnaire completion rates are reported, no information is provided about reasons for non-completion or whether non-responders differed systematically from those who did respond. If such information is available, I would ask that this is reported. If not available, I feel this should be states as a limitation and the authors should offer some consideration of the possible implications for interpretation (e.g., potential non-response bias).

5. At present there appears to be no consideration of the potential limitations of the research. I feel the authors should acknowledge these as this will strengthen the reader's ability to interpret the findings and their relevance to future trials.

Minor points

- There is a missing reference cited on line 157 — please add or correct this citation.

VERSION 1 - AUTHOR RESPONSE

Reviewer feedback	Response by authors
Reviewer 1	*Note: line numbers refer to those in the revised manuscript with tracked changes.*
Abstract	
1. Results: When reporting the results, for clarity it would be beneficial if it was clear which data represents data from participants and which (if any) is from the health coaches. Both are documented as being involved in the 'outcome measures' section.	Most of the responses referred to are from participants. Hence, we have added a comment on p.8 stating, 'Data refer to participants rather than the health coaches, unless indicated'. See Line 188.
2. Line 28-29: Appreciate there are word count restrictions, but it would be beneficial to make it clearer what 'study evaluation methods' were that could have influenced behaviours, as these are not documented in preceding sections of the abstract.	The statement we provided has been modified. We have provided a qualifying statement: (e.g., activity measures increasing awareness and execution of behaviours). See Line 29.
Introduction	
3. Lines 48-50: It would be beneficial to make clear that sitting is a form of sedentary behaviour. The first sentence describes sedentary behaviours, then the next describes breaking up sitting, but how these behaviours are linked is not made clear.	We have added a brief definition of sedentary behaviour, with citation. See Line 52.
4. Line 69: It would be more appropriate and representative of the aim of the intervention if this was described as 'break up periods of sitting' rather than 'inactivity'	We have reworded this to reflect sedentary behaviour rather than inactivity. Thanks for the good suggestion. See Line 74.
5. Lines 70-72: It would be beneficial to make it clear here who specifically found the interview to be feasible and acceptable i.e., was it participants and health coaches?	We have clarified this by adding 'to participants'. Please see Line 76.
Methods	
6. General: As this later emerges in interview data, it would be valuable to describe when the study was conducted (i.e., dates) and when the intervention took place making reference to the health landscape at this time i.e., it was during a global pandemic.	We have added: The study took place from October 2020 to November 2021 in England, hence was conducted during the COVID-19 pandemic with lockdown restrictions often in place. See Lines 111-113.
7. Line 98: Please rephrase to 'were involved with the delivery of'	This has been edited to state: The health coaches involved with delivery of RESIT were also interviewed. See Line 110.
8. Questionnaires: Can the authors provide any details regarding how the items of the questionnaire were developed? It is noted that in the paper cited as reference number 15, the methods describes that 'A process evaluation questionnaire that was developed following Medical Research Council guidelines [35] and previous research [36]' – if this	We have added a statement to describe how the questionnaires were developed as suggested: "The questionnaires were developed following Medical Research Council guidelines and informed by previous research". See lines 126-127.

Reviewer feedback	Response by authors
describes what is reported in this study, this detail should be included	
9. Interviews: Please describe where these were conducted i.e., the environment for in-person interviews or if online	We have added that interviews were conducted online or by telephone. See Line 138-139.
10. Interviews: Was the interviewer (MLB) involved in any other stages of the project delivery or engagement with participants with the intervention? It would be important to make this clear to understand if participant's responses could be biased by their involvement in the intervention or not	We have added the following statement accordingly: "MLB was involved in delivery of the project (participant recruitment, intervention facilitation and outcome measure evaluation)." See lines 139-140.
11. Line 129: Can the sex characteristics and mean age of the health coaches be included, as has been reported for the intervention and control group participants	We have added that 80% of the health coaches were female (line 156). We did not collect data on age and therefore cannot include this.
12. Lines 147-148: Please consider reviewing the phrase 'NVivo for analysis' since this supports researchers to organise, code, and interpret qualitative data, but the analysis itself is conducted by the researcher not the software	We agree! Apologies for the poor phraseology. This has been edited to: support with data coding and organising. See Line 176.
13. Line 157: A reference is needed where (ref) is currently written	Now added.
14. Patient and Public Involvement: Did the researchers work with the PPI group in any way to develop the interview schedule? Were any pilots of the interview schedule or survey conducted to ensure comprehension?	The interview schedule was not developed with patient and public involvement. It was informed by previous research (Edwardson et al., 2018 – manuscript ref #19) and involved some of the current authors, and is published in the Findings paper in Supplementary Material 2. It was not piloted due to previous experience.
Results	
15. Lines 172: It would be beneficial to make it clear here at the start of this section if themes are derived from interviews with participants and/or health coaches.	We have added the statement: Results are for participants unless otherwise indicated. See Line 188-189. See also Line 211.
16. Line 185: It would be beneficial to make it clear here this represents interview data with the participants (i.e., not also the health coaches)	See comments above.
17. Reasons for participation: It would be beneficial to make it clearer specifically what other sub-themes were. One is named as 'health motivation' but others are not explicitly stated. For example, it is assumed 'additional time' and 'altruism' are other sub-themes?	We have added this text: Sub-themes included health motivation, altruism, and having the time. See Line 216.
18. Line 210: For clarity, it would be beneficial to specify this data is from the questionnaires.	This has been clarified by adding the statement: who responded to the questionnaire. See Line 241.

Reviewer feedback	Response by authors
19. Line 220: Please revise this statement to align with terminology used in Supplementary Table 4. The rows in the table describe ‘5 extremely useful, 4 very useful, 3 moderately useful, and 2 somewhat useful’. In the text, it describes ‘useful or extremely useful. Does ‘useful’ incorporate all statements 2 – 4?<br><br>Furthermore, to support this statement in the manuscript itself, could the authors provide a summary/mean/range of percentage values to quantify what they mean by ‘nearly all’.	The statement has been revised. See Line 251.<br><br>We have now edited the text read: The majority of participants considered these elements to be at least ‘moderately useful’ (85-100% across elements) (Supplementary Table 4). See Line 250.
20. Lines 230-232: Is this from data that has been reported previously? If so, it should be made clear	No. These data are reported here for the first time.
21. Lines 264: Please make it clear if when the intervention group ‘were asked’ if this is via interview or questionnaire data collection methods	We have added via questionnaire. See Line 296.
22. Lines 384-397: This sentence feels more of a discussion of the findings, rather than reporting of results	We have now removed this sentence and further developed the discussion around this from line 573: “This could include the provision of alternative furniture (e.g., sit-to-stand desks) to enable the same work to continue whilst standing and opportunities to sit less during work (e.g., regular breaks or other regulatory, social, and environmental changes that encourage or prompt less sitting)”
23. Lines 395: Please make it clear if when the intervention group ‘were asked’ if this is via interview or questionnaire data collection methods	We have added: via open-ended responses in the questionnaire. See Line 427.
Discussion	
24. Line 464: Has health coaching always been delivered remotely or has this also been via in-person sessions? This could be an important discussion point if similar benefits/engagement have been reported by participants, despite the remote nature of delivery	Both in-person and remote delivery approaches have been effective in previous research. We have now stated: Health coaching has been used widely, including remote delivery, to modify lifestyle in adults with T2DM with resultant improvements in glucose control. See lines 496.
25. Lines 477-478: Do the authors have a reference to support this statement regarding the rapid expansion of e- and m-health interventions? Also, whilst it may seem obvious, for clarity, it would be beneficial to explain why there is an ‘inevitable’ increase in the use of these types of intervention strategy.	We have added a reference and deleted the word ‘inevitably’. See Line 511.

Reviewer feedback	Response by authors
26. Lines 484-485: Can the authors offer any thoughts on why the computer software was less adopted? Is this in line with any other research assessing computer software interventions to reduce sitting time, or research comparing computer software interventions and wearables?	We have added the statement: Computer software being less mobile could be one explanatory factor, especially for participants who were not office workers. See Line 519.<br><br>We were unable to locate literature comparing the use of computer software and wearables so are unable to discuss the findings in this context. We have recommended in this section that wearable and smartphone apps are used in future interventions that aim to reduce sitting in community settings. See p20.
27. Lines 489-490: It would be beneficial to align this point to the current intervention, i.e., RESIT used a combination of strategies and has been successfully received by participants	Thank you for this suggestion. We have added: These findings are supported by the present study, which demonstrated the use of a combination of strategies was successfully received by participants. See line 524.
28. Lines 537-539: It is important to note with this statement that whilst environmental changes may be 'obvious' they may not always be possible in certain work environments. For example, the cost of alternative furniture may not be feasible for certain workplaces. Furthermore, permitting high frequency breaks may not be permissible in environments such as contact centres or customer-facing roles.	This is a good point. We have added: Notwithstanding such potential solutions, it should be noted that some jobs may preclude taking breaks or sitting less, and some environmental solutions may be costly. See Line 576.
29. Line 565: It would be important to mention in this section regarding reasons for taking part that the COVID-19 pandemic, when this study was completed, may have also had an impact. In the results section participants reported having more time to take part due to COVID-19 and working from home/having more time– which may be less likely in a future implementation of the intervention as restrictions have now eased. Furthermore, could participant's awareness of the need to contribute to health research have been heightened due to the pandemic and associated news stories around health/health-related research?	These are excellent points. We have added: It is also possible that the study taking place during the COVID-19 pandemic influenced reasons for taking part, such as greater awareness of health and more time to take part. See Line 609.
Reviewer 2	
1. I note that the interventions theory of change is not reported on therefore no rationale for the focus of the research questions being addressed is provided. Given the theory of change should guide which mechanisms and processes are examined, I	We thank the reviewer for this thoughtful comment and fully appreciate the importance of theory-driven process evaluations. However, the authors feel that inclusion of a theory of change or a logic model is not appropriate within the

Reviewer feedback	Response by authors
recommend the authors include a succinct description (and ideally a figure or logic model) that sets out the hypothesised causal pathways and the specific mechanisms the evaluation sought to test.	current manuscript. The RESIT intervention's theoretical underpinnings have already been reported in the protocol paper, which is cited in this manuscript.<br><br>The current manuscript explores the processes through which the trial and intervention were implemented and experienced, focusing on parameters necessary to inform a future full-scale trial, rather than on mechanism testing. As such, the research questions are aligned with process evaluation objectives rather than causal mechanisms. We therefore feel it would be misleading to present a full theory-of-change model implying a level of mechanism testing that is beyond the scope of this paper and the current phase of research.
2. Although a well-written aim is provided, on reading the manuscript in its entirety I feel it does not fully deliver on this aim as not all of the findings from the process evaluation are reported on. Whilst the results section transparently states that other findings are reported elsewhere and cites that work, I feel this manuscript would benefit from a brief justification for reporting findings across separate papers. I also wonder if the aim should be re-written to reflect the narrower scope of this manuscript?	We have now amended the latter part of the introduction ("Intervention acceptability, fidelity and engagement are reported elsewhere alongside outcomes related to feasibility and acceptability of the trial") and aim to make clear the reporting of other process evaluations in a separate paper and the specific aim of this paper with respect to reporting "the process evaluation findings to understand, in-depth, how a tailored intervention (RESIT) to reduce and break up sitting in individuals with T2DM operated and was experienced during a randomised controlled feasibility trial". See from line 93.
3. I feel the authors need to provide a clear rationale for interviewing only a subset of participants (including how the subset was selected) and provide comment on whether those interviewed were representative of the trial sample. I would also ask that the authors include details on the sampling strategy (e.g., purposive for variation, convenience, or pragmatic) and any demographic differences between interviewees and non-interviewees that might influence interpretation.	In the Protocol paper (manuscript ref # 15), we stated semi-structured interviews would be completed with a subset of intervention participants. An *a-priori* sample of 13 interviews for each study arm was deemed sufficient to uncover common patterns and themes (Francis et al., 2010, *Psychology and Health*, 25(10), 1229– 1245) (as reported in the Trial Findings paper). Based on previous research, the rationale for interviewing a subset of participants is data saturation (i.e., when no new information is coming out, when further coding is no longer possible, and there is

Reviewer feedback	Response by authors
	enough information to replicate the study) (Fusch & Ness, 2015, *The Qualitative Report,* 20(9): 1408-1416). In the end, we overrecruited by inviting all participants and this was ultimately necessary to be able to speak with people in depth about their experiences.<br><br>See Line 140: Participants (n=65) were invited at random to take part in the interviews following completion of their 6-month measurements. Of those, 45 agreed to interview (20 Control and 25 Intervention) with 31 booking an interview (15 Control, 16 Intervention) and 25 attending an interview (12 Control, 13 Intervention).<br><br>Regarding representativeness, we have added text: We spoke with 37% of intervention participants (n=13 of 35 starting at baseline) and 34% of controls (n=12 of 35). Raw demographic characteristics of all interviewees have been reported previously in Supplementary Material 4 of the Findings paper. Demographic characteristics of those interviewed were very similar to the corresponding study arm (see Supplementary Table 2). See from Line 143.
4. Although questionnaire completion rates are reported, no information is provided about reasons for non-completion or whether non-responders differed systematically from those who did respond. If such information is available, I would ask that this is reported. If not available, I feel this should be states as a limitation and the authors should offer some consideration of the possible implications for interpretation (e.g., potential non-response bias).	Data not available. A statement has been added to the limitations. See p24.
5. At present there appears to be no consideration of the potential limitations of the research. I feel the authors should acknowledge these as this will strengthen the reader's ability to interpret the findings and their relevance to future trials.	We have added comments on limitations. Some of these were already there, but we have attempted to make this clearer and have developed these further. See p24.
6. There is a missing reference cited on line 157 — please add or correct this citation	Corrected.

VERSION 2 - REVIEW

Reviewer 1
Name Carter, Sophie E
Affiliation York St John University
Date 09-Jan-2026
COI

Thank you for the authors for taking the time to respond to the feedback from reviewers and amend their manuscript accordingly. All comments have been satisfactorily addressed.

Reviewer 2
Name Airlie, Jennifer
Affiliation Durham University, Department of Sport and Exercise Sciences
Date 26-Jan-2026
COI

Thank you for considering all the suggestions I put forward in my initial review and addressing these. I appreciate the authors efforts to revise the manuscript.

As a minor point, I have noted that in an effort to perhaps address a point I raised in my initial review (point 3) I feel there is now some duplication of information in the 'Interview' sub-section of the manuscript. I would suggest the authors look at lines 141 – 147 and lines 152 – 156 specifically and delete / reword where appropriate.

In addition, whilst I note and appreciate that the authors are clear that the intention of the RESIT intervention was to reduce sedentary time in ambulatory individuals, and an ability to be able to ambulate was one of the eligibility criteria for the trial, I would like to see some acknowledgement that this perhaps precludes the generalisability of the findings. Related to this, I would encourage the authors to reflect and perhaps re-consider the use of phrases such as "sit less" and considering using more inclusive language such as "Don't be still for too long". Apologises I did not pose this suggestion in my initial review, but I have pondered this since I first read the manuscript and it is something that came to mind again when re-reading. I therefore felt it was important to bring this to the attention of the authors. Given the intervention name foregrounds the regulation of sitting time rather than simply a reduction, I feel the authors do have an appreciation of the importance of language in this space and hope they will give some thought my suggestion.

VERSION 2 - AUTHOR RESPONSE

Reviewer: 1
Dr. Sophie E Carter, York St John University
Comments to the Author:
Thank you for the authors for taking the time to respond to the feedback from reviewers and amend their manuscript accordingly. All comments have been satisfactorily addressed.

Response: Thank you to the reviewer for reviewing our revised manuscript.

Reviewer: 2
Dr. Jennifer Airlie, Durham University
Comments to the Author:
Thank you for considering all the suggestions I put forward in my initial review and addressing these. I appreciate the authors efforts to revise the manuscript.

As a minor point, I have noted that in an effort to perhaps address a point I raised in my initial review (point 3) I feel there is now some duplication of information in the 'Interview' sub-section of the manuscript. I would suggest the authors look at lines 141 – 147 and lines 152 – 156 specifically and delete / reword where appropriate.

Response: Thank you to the reviewer for reviewing our revised manuscript and spotting this duplication of information. This has been revised to remove the repetition within lines 131-145.

In addition, whilst I note and appreciate that the authors are clear that the intention of the RESIT intervention was to reduce sedentary time in ambulatory individuals, and an ability to be able to ambulate was one of the eligibility criteria for the trial, I would like to see some acknowledgement that this perhaps precludes the generalisability of the findings. Related to this, I would encourage the authors to reflect and perhaps re-consider the use of phrases such as "sit less" and considering using more inclusive language such as "Don't be still for too long". Apologises I did not pose this suggestion in my initial review, but I have pondered this since I first read the manuscript and it is something that came to mind again when re-reading. I therefore felt it was important to bring this to the attention of the authors. Given the intervention name foregrounds the regulation of sitting time rather than simply a reduction, I feel the authors do have an appreciation of the importance of language in this space and hope they will give some thought my suggestion.

Response: This is aa valuable point raised by the reviewer and we have acknowledged the potential limitation with respect to generalisability in our limitations section. We have also made suggestions regarding terminology that can be considered depending on the intended target of such interventions. Please see lines 615-620:

"Regarding the generalisability of our trial, it is worth recognising the importance of language. We restricted our 'sit less' intervention and messages to those who were

ambulatory. Future studies may wish to broaden this focus and include those who are non-ambulatory or have other movement conditions. If so, messaging around spending less time 'still' or 'sedentary', rather than 'sitting', may be appropriate”.
